# Behavioral and neuropathological characterization over the adult lifespan of the human tau knock-in mouse

**DOI:** 10.3389/fnagi.2023.1265151

**Published:** 2023-09-29

**Authors:** Matthew J. Benskey, Spencer Panoushek, Takashi Saito, Takaomi C. Saido, Tessa Grabinski, Nicholas M. Kanaan

**Affiliations:** ^1^Department of Translational Neuroscience, College of Human Medicine, Michigan State University, Grand Rapids, MI, United States; ^2^Department of Neurocognitive Science, Institute of Brain Science, Nagoya City University Graduate School of Medical Sciences, Nagoya, Japan; ^3^Laboratory for Proteolytic Neuroscience, Riken Center for Brain Science, Wako, Japan; ^4^Neuroscience Program, Michigan State University, East Lansing, MI, United States

**Keywords:** tau, MAPT, aging, knock-in mice, glial cells, mouse model, tauopathy, Alzhiemer’s disease

## Abstract

Tau is a microtubule-associated protein with a diverse functional repertoire linked to neurodegenerative disease. Recently, a human tau knock-in (MAPT KI) mouse was developed that may overcome many limitations associated with current animal models used to study tau. In MAPT KI mice, the entire murine *Mapt* gene was replaced with the human *MAPT* gene under control of the endogenous *Mapt* promoter. This model represents an ideal *in vivo* platform to study the function and dysfunction of human tau protein. Accordingly, a detailed understanding of the effects *MAPT* KI has on structure and function of the CNS is warranted. Here, we provide a detailed behavioral and neuropathological assessment of MAPT KI mice. We compared MAPT KI to wild-type (WT) C57BL/6j mice in behavioral assessments of anxiety, attention, working memory, spatial memory, and motor performance from 6 to 24 months (m) of age. Using immunohistological and biochemical assays, we quantified markers of glia (microglia, astrocytes and oligodendrocytes), synaptic integrity, neuronal integrity and the cytoskeleton. Finally, we quantified levels of total tau, tau isoforms, tau phosphorylation, and tau conformations. MAPT KI mice show normal cognitive and locomotor behavior at all ages, and resilience to mild age-associated locomotor deficits observed in WT mice. Markers of neuronal and synaptic integrity are unchanged in MAPT KI mice with advancing age. Glial markers are largely unchanged in MAPT KI mice, but glial fibrillary acidic protein is increased in the hippocampus of WT and MAPT KI mice at 24 m. MAPT KI mice express all 6 human tau isoforms and levels of tau remain stable throughout adulthood. Hippocampal tau in MAPT KI and WT mice is phosphorylated at serine 396/404 (PHF1) and murine tau in WT animals displays more PHF1 phosphorylation at 6 and 12 m. Lastly, we extended previous reports showing that MAPT KI mice do not display overt pathology. No evidence of other tau phosphorylation residues (AT8, pS422) or abnormal conformations (TNT2 or TOC1) associated with pathogenic tau were detected. The lack of overt pathological changes in MAPT KI mice make this an ideal platform for future investigations into the function and dysfunction of tau protein *in vivo*.

## Introduction

Tau is a microtubule-associated protein that is predominantly expressed in the nervous system where it plays a role in regulating cytoskeletal dynamics and signaling in the axon (Kanaan and Grabinski, 2021; Mueller et al., 2021). Beyond these well described functions, tau is implicated in a broad range of cellular functions within different cellular compartments of neurons and glia (Wang and Mandelkow, 2016). As such, research into the physiological function of tau protein is an area of active research. Tau is also implicated in the pathogenesis of many neurodegenerative diseases. Under pathological conditions, tau can form insoluble aggregates that are a hallmark of diseases collectively referred to as tauopathies, including Alzheimer’s Disease (AD), progressive supranuclear palsy (PSP), frontotemporal dementia with parkinsonism linked to chromosome 17 (FTDP-17), corticobasal degeneration (CBD), argyrophilic grain disease, Pick’s disease and chronic traumatic encephalopathy (CTE) (Lee et al., 2001; Braak et al., 2006; Dickson et al., 2011; Kouri et al., 2011; Irwin et al., 2016; Kovacs et al., 2020). Based on the critical role tau plays in normal neurophysiology and the severe consequences of tau dysfunction, there is a great need for a detailed understanding of the role tau plays in health and disease.

Much of the currently available knowledge regarding the physiological function of tau and mechanisms of tau toxicity were obtained through pre-clinical animal models. Most of the existing models provide an opportunity to study tau-related toxicity using transgenic overexpression of tau and/or the function and dysfunction of endogenous murine tau. Within tau transgenic models, a human tau transgene encoding a single isoform of tau, usually containing an FTDP-17-linked mutation, is expressed at supraphysiological levels within the CNS. The overexpression of tau results in aggregation and subsequent neurodegeneration (Ramsden et al., 2005; Yoshiyama et al., 2007). Limitations of these models include use of tau overexpression (typically 5- to 13-fold) (Ramsden et al., 2005; SantaCruz et al., 2005; Yoshiyama et al., 2007), disruption of endogenous genes following insertional mutagenesis of the transgene (Gamache et al., 2019; Goodwin et al., 2019), and use of mutant tau. In addition to traditional transgenics, a human tau mouse was created using a PAC to express the human *MAPT* gene on a mouse tau knockout background. These mice lack murine tau and overexpress all 6 human tau isoforms, resulting in neuropathological and cognitive deficits in later ages (Andorfer et al., 2003, 2005; Polydoro et al., 2009; Phillips et al., 2011). These models have helped improve our understanding of tau-related disease mechanisms and with testing potential therapeutics; however, all have important limitations.

The other primary strategy used to study tau physiology and pathophysiology focuses on manipulating endogenous murine tau in wild type (WT) animals. The advantages of these models are the use of unmodified WT tau expressed at physiological levels. However, the major limitations are differences between human and murine tau. Within the adult human brain, alternative splicing of the *MAPT* gene results in the formation of mainly 6 tau isoforms (Goedert et al., 1989). Tau isoforms differ in the presence or absence of two possible N-terminal insertions (designated 0 N, 1 N, or 2 N) and whether they contain three or four microtubule binding repeat domains (designated as 3R or 4R). The adult human brain contains roughly equal proportions of 3R and 4R tau isoforms (Goedert et al., 1989; Kosik et al., 1989). In contrast, the adult murine brain solely expresses 4R isoforms. Further, human tauopathies are categorized based on the tau isoforms found within aggregates. More specifically, tauopathies are classified as 3R tauopathies (Pick’s disease), 4R tauopathies (CBD, PSP, AGD), or 3R + 4R tauopathies (AD, CTE) (Lee et al., 2001). Thus, research into mechanisms of tau function and dysfunction based solely on 4R murine tau cannot recapitulate the full diversity of tau isoforms found in humans. Finally, there are also significant differences in the primary amino acid sequence between murine and human tau. Of note, human tau contains an N-terminal 11 amino acid sequence that is completely absent in murine tau as well as many individual amino acids within the N-terminus that differ from murine tau (Hernández et al., 2020). The N-terminal amino acid insert facilitates interaction with synaptic proteins and is hypothesized to affect tau intramolecular folding, axonal transport, and cellular energy metabolism (Hernández et al., 2020; Mueller et al., 2021). Taken together, the differences in sequence and isoform expression between human and murine tau limit the utility of findings gained from the study of murine tau.

Recently, the Saido group published the description of a human MAPT knock-in (MAPT KI) mouse that overcomes many of the limitations of available tau models (Hashimoto et al., 2019; Saito et al., 2019). Within this mouse, the entire murine *Mapt* gene was replaced with the human *MAPT* gene and is expressed under control of the endogenous murine *Mapt* promoter. MAPT KI mice express all six isoforms of tau in a ratio roughly equal to that of the adult human brain and show a normal subcellular localization of tau (Hashimoto et al., 2019; Saito et al., 2019). Humanization of the murine *Mapt* gene did not impair Y-maze performance, produce gross structural changes in the brain (as quantified by T2 weighted MRI), increase amyloid-β deposition or modify markers of apoptosis and gliosis (Hashimoto et al., 2019; Saito et al., 2019). While these studies provided important initial descriptions of MAPT KI mice, analyses at additional ages and measures that may detect more subtle changes in nervous system structure (such as changes in synaptic integrity) would benefit the field.

Due to the tremendous utility this model holds for the tau field, we believe it is essential to have an in-depth characterization of the MAPT KI mouse phenotype throughout their adult lifespan. Thus, the purpose of the current study was to provide a detailed behavioral and neuropathological assessment from 6 to 24 months (m) of age in male and female MAPT KI mice. To this end, we performed behavioral assessments of anxiety, attention, working memory, spatial memory, and motor performance over the adult lifetime of MAPT KI mice. Using both histology and biochemistry we quantified markers of glia (microglia, astrocytes and oligodendrocytes), synaptic integrity, neuronal integrity and the microtubule system in adult MAPT KI mice. Finally, we extended previous reports by quantifying total tau expression, levels of tau isoforms, tau phosphorylation, and tau conformation over the adult lifetime of MAPT KI mice. This information can serve as a baseline comparison for future studies utilizing MAPT KI mice to study the tau protein.

## Methods

### Animals

Breeding pairs of MAPT KI mice were obtained from Dr. Karen Duff at Columbia University with permission from the Saido group at the Riken Center for Brain Science. Breeding pairs were used to generate an in-house colony. For MAPT KI breeding, a single adult male mouse was housed with two adult female mice (all homozygous for MAPT KI). Following birth of new litters, offspring were weaned from the Dam at day P20-P21. Experimental groups formed from in-house colonies were composed of animals from multiple litters. Adult male and female wildtype C57/BL6j (Jackson Labs, stock #000664) were obtained from Jackson labs at 6, 12, 15 or 20 m of age. The 15 m and 20 m-old animals were then aged to 18 m and 24 m, respectively, in our animal facility prior to testing. Animal weights at 6, 12, 18 and 24 m are shown in Supplementary Figure S1. All mice were housed in a 12-h light/dark cycle with food and water provided *ad libitum*. All studies were conducted in compliance with federal, state, and institutional guidelines and approved by the Michigan State University Institutional Animal Care and Use Committee.

### Behavioral testing

Individual groups of male and female MAPT KI and WT animals underwent a battery of behavioral tests at 6, 12, 18, and 24 m of age. Behavioral testing was performed at the same time of day for all cohorts. For all behavioral tests, animals were singly housed prior to the task and allowed to acclimate in the room containing the behavioral apparatus for at least 30 minutes (min) prior to testing. The room containing the behavioral apparatus was partially sound proofed and a white noise machine was used to eliminate extraneous noises that could affect the experiment. The experimenter was blinded to all treatment groups throughout the entirety of the behavioral testing and analysis.

#### Open field

Animals were placed individually within a 40 cm^2^ open field apparatus box with a video camera overhead. AnyMaze video tracking software (Stoelting Co.) was used to delineate the center (~28 cm^2^ square center of the apparatus) and peripheral (~6 cm wide zone surrounding the center zone) zones within the open field apparatus. AnyMaze software was used to track the movements of the animal’s head during a single 15 min trial. AnyMaze software was used to quantify total time in each zone as well as total distance traveled. Between each trial the behavioral apparatus was cleaned with 70% ethanol and allowed to completely dry to eliminate residual olfactory stimuli.

#### Object location task (OLT) and novel object recognition task (NORT)

**Figure 1:**
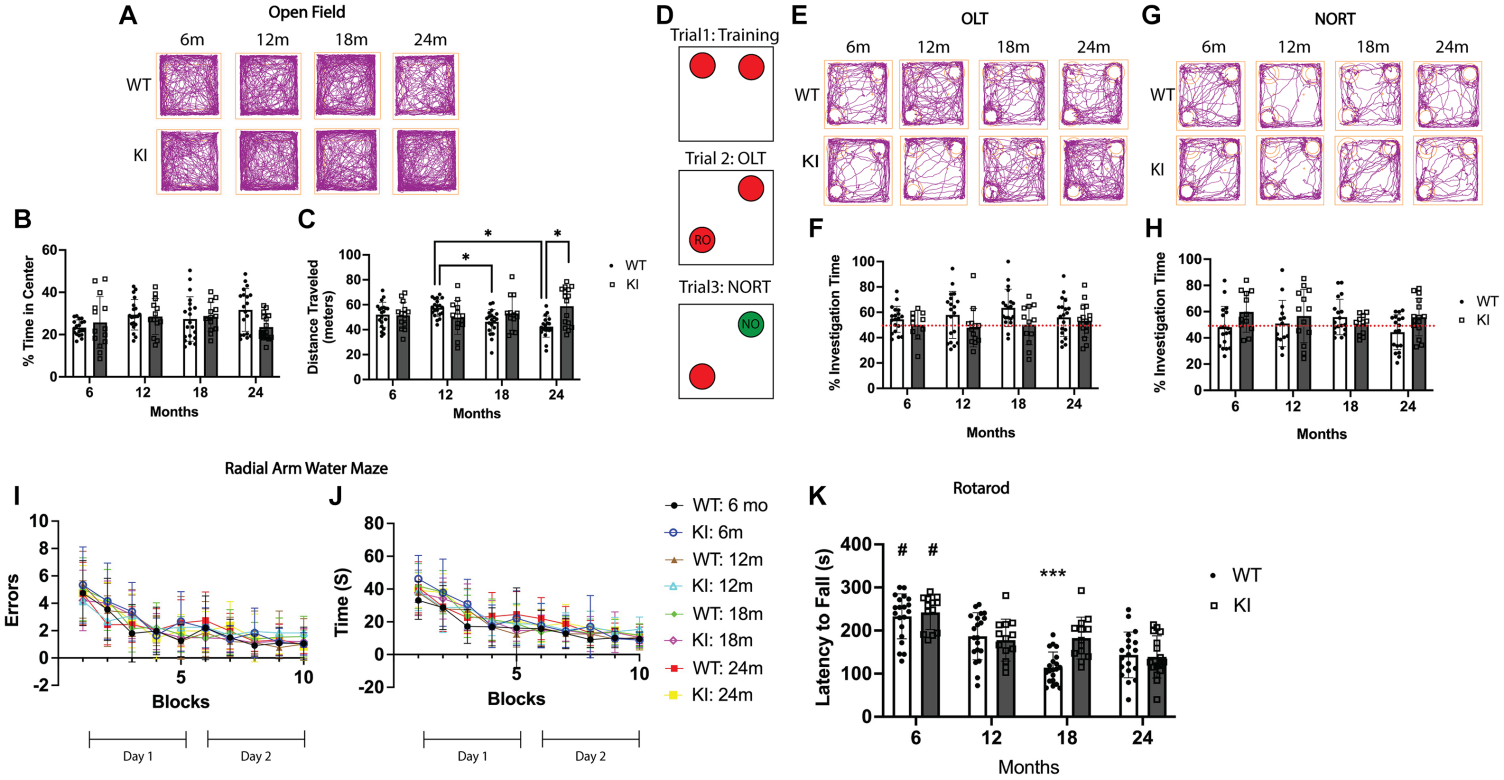
Behavioral assessment of MAPT KI and WT mice. Male and female human MAPT knock-in (MAPT KI or KI) and wild type (WT) mice completed a battery of behavioral tests at 6, 12, 18, and 24 months (m) of age (*n* = 6–10/group). For all tasks, no sex differences were detected within a given age and genotype (see Supplementary Table S1 for data on individual sexes). Sexes were combined within age and genotype for all subsequent analyses. **(A)** Representative track traces from the open field test. **(B)** Quantification of the percent of time spent within the center square of the open field apparatus. **(C)** Quantification of total distance traveled (in meters) in the open field apparatus. 24 m WT mice traveled significantly less distance during the open field test than 24 m MAPT KI mice, and the 12 m WT mice. 18 m WT mice traveled significantly less distance than 12 m WT mice. **(D)** Schematic illustration of the object location task (OLT) and novel object recognition task (NORT). Briefly, mice were trained to recognize two identical objects in trial 1. In trial 2 (OLT), one of the objects is moved to a new corner. Time investigating the relocated object (RO) is quantified. In trial 3 (NORT), the object that was not moved in trial 2, is replaced with a novel object (NO) and time spent investigating the novel object is quantified. **(E)** Representative track traces from the object location task. **(F)** Quantification of percent investigation time of the relocated object in trial 2. **(G)** Representative track traces from the novel object recognition task. **(H)** Quantification of percent investigation time of the novel object in trial 3. **(I)** Quantification of the number of errors made prior to reaching the escape platform during the radial arm water maze. **(J)** Quantification of latency to reach the escape platform in the radial arm water maze. **(K)** Quantification of latency to fall from the rod during the rotarod task. MAPT KI and WT mice showed an age-related decline in rotarod performance at 12, 18, and 24 m when compared to 6 m genotype matched animals. 18 m WT mice also spent significantly less time on the rod compared to 18 m KI animals. For all histograms, individual data points are shown while bars represent mean of groups ± standard deviation. * *p* ≤ 0.05. # Significantly different than all other ages (*p* ≤ 0.05). *** Significantly different than 18 m MAPT KI and 12 m WT and MAPT KI (*p* ≤ 0.05) (*n* = 12–20 for combined sex groups).

OLT-NORT was performed as described in (Denninger et al., 2018) with minor modifications. The OLT-NORT task was broken into 3 × 10-min trials (trail 1-training; trial 2-OLT; trial 3-NORT) with a 20-min inter-trial interval between the trials (Figure 1D). For all trails, animals were placed individually in a 40 cm^2^ behavioral testing box with a video camera mounted above the box. Spatial cues were present on three of the walls of the behavioral apparatus to allow the animals to orient themselves during the task. During each trail, AnyMaze video tracking software was used to track the movement of the animal’s head and the amount of time the animals spent investigating each object (as indicated by time within object zones that were delineated within the AnyMaze software). Trial 1-training: animals were placed into the behavioral apparatus that contained 2 identical objects located in the two back corners of the apparatus (~ 6 cm away from each wall). Animals were allowed to explore the objects for the 10-min trial and AnyMaze software was used to quantify the amount of time the animals spent exploring each object. Trial 2-OLT: One of the objects used in trial 1 was moved to a new corner (again ~6 cm away from each wall) while the other object used in trial 1 remained in the same position. Animals were allowed to explore the objects for the 10-min trial and time spent exploring each object was quantified. Trial 3-NORT: The object that was not moved in trial 2, was replaced with a novel object. Animals were allowed to explore the objects for the 10-min trial and time spent exploring each object was quantified. In both OLT and NORT, *a priori* criteria were set such that mice must have a total exploration time of at least 20 s to be included in the analysis (Denninger et al., 2018). Between each trial the behavioral apparatus was cleaned with 70% ethanol and allowed to dry to eliminate any residual olfactory stimuli.

#### Rotarod

Motor performance was assessed with the rotarod task. The rotarod task takes place over two consecutive days. On the first day, animals underwent four training trials. On the second day, animals underwent four trials that were averaged to give the mean latency to fall from the rod. Animals were split into cohorts of four that completed trials at the same time. Each cohort consisted of animals from different experimental groups. Within each trial, all animals within a cohort were placed onto their individually separated rods in the rotarod module for mice (TSE-Systems Inc.). Once all animals were loaded into the rotarod module, the protocol was initiated. The protocol begins at 4 RPMs and accelerates to a final speed of 400 RPMs over a 300 s time frame. The latency to fall for each animal was recorded. Between each trial the rotarod module was cleaned with 70% ethanol and allowed to dry to eliminate any residual olfactory stimuli.

#### Radial arm water maze (RAWM)

Memory deficits were investigated using the RAWM as previously described (Alamed et al., 2006). The RAWM utilizes a circular pool separated into six arms. Water in the pool is kept at 26° Celsius (C). The pool is surrounded by spatial cues on the walls that allow animals to orient themselves during the task. An escape platform is put at the end of one arm, and each animal is assigned a different escape arm that remains constant throughout the entirety of the task. Animals are placed into the pool at the end of one of the non-escape arms and allowed to swim for up to 1 min to find the escape platform. During this time the latency to reach the platform as well as the number of errors is recorded. An error consists of entering any arm other than the escape arm or the failure to make a decision (staying in any non-escape arm for more than 15 s). Once the animal finds the escape platform it is allowed to rest on the platform for 20 s to establish the location of escape platform relative to the spatial cues. On subsequent trials the start arm is varied so that the animal must utilize the spatial cues to guide itself to the platform. The RAWM task is split into 2 days with each day consisting of 15 trials. On day one, the escape platform is alternated between a visible platform (which breaches the water so the animal can see it) and a hidden platform (which is completely submerged under ~3 mm of water) between each trial. On day two, the escape platform is always hidden. Following six trials, animals were allowed to rest for at least 30 min before completing subsequent trials. The average number of errors from three trials are binned into blocks, and data from the 10 blocks are reported.

### Tissue collection and processing

At the age of sacrifice, mice were administered an overdose of Fatal Plus solution (≥100 mg/kg).

#### For histology

Mice were transcardially perfused with 0.9% saline containing heparin (10,000 units/L) followed by 4% paraformaldehyde. Brains were then removed and post-fixed in 4% paraformaldehyde overnight. For cryoprotection, brains were immersed first in a 15% sucrose solution until saturated, followed by a 30% sucrose solution until saturated. A freezing stage, sliding knife microtome was used to prepare six separate series of 30 μm coronal brain sections encompassing the entire rostral caudal axis of the brain.

#### For biochemistry

Mice were transcardially perfused with 0.9% saline containing heparin (10,000 units/L). Brains were then removed, and the hippocampus was immediately dissected and frozen on dry ice.

### Immunohistochemistry

Immunohistochemistry was performed using standard protocols (Kanaan and Grabinski, 2021). Briefly, fixed coronal sections were washed 6 × 10 min in tris-buffered saline containing 0.5% triton X-100 (TBS-Tx). Endogenous peroxidase activity was quenched by incubating tissue for 1 h in 3% hydrogen peroxide diluted in TBS-Tx, followed by 6 × 10 min washes in TBS-Tx. Tissue was blocked for 1 h in TBS-Tx containing 10% goat serum and 2% bovine serum albumin. Tissue was then incubated overnight at 4° C in primary antibody diluted in TBS-Tx containing 2% goat serum. Primary antibodies used for immunohistochemistry were biotinylated AT8 (1:1,000; Thermo, MN1020B; RRID: AB_223647), pS422 (1:2,500; Abcam, ab79415, RRID: AB_1603345), biotinylated PHF1 (1:100; RRID: AB_2315150), biotinylated TNT-2 (1:100; RRID: AB_2736931), and biotinylated TOC1 (1,1,000; RRID: AB_2832939). Following primary antibody incubation tissue was washed 6 × 10 min in TBS-Tx. After which, the tissue series labeled with non-biotinylated primary antibodies were incubated for 2 h at room temperature in goat anti-rabbit biotinylated (Vector Laboratories, BA-1000; RRID: AB_2313606) secondary antibodies diluted at 1:500 in TBS-Tx containing 2% goat serum, followed by 6 × 10 min washes. All tissue was then incubated for 2 h in ABC Elite solution (Vector Labs, PK6100). Following 6 × 10 min washes in TBS-Tx, reactivity was visualized using 3, 3′-diaminobenzidine (Sigma, D5637) at 0.5 mg/mL in TBS-Tx with 0.003% hydrogen peroxide for 5–10 min. Tissue was then washed in TBS, mounted on microscope slides and processed through ethanol and xylenes before cover slipping with Cytoseal-60 (Thermo, 831016). Images were acquired with a Nikon Eclipse 90i microscope, a Nikon DS-Ri1 camera and Nikon Elements Software (Nikon Instruments, Melville, NY). All individual images between animals within a particular stain were acquired using identical microscope and post-processing parameters (magnification, light source intensity, exposure time and contrast). Images were prepared for publication using Adobe Photoshop and Illustrator.

### Immunofluorescence and quantification

Immunofluorescence staining using LiCor near-infrared secondary antibodies was performed using previously established protocols (Benskey et al., 2016, 2018). Fixed coronal sections were washed 6 × 10 min in TBS-Tx and blocked for 1 h in TBS-Tx containing 10% goat serum and 2% bovine serum albumin. Tissue was then incubated overnight at 4°C in primary antibody diluted in TBS-Tx containing 2% goat serum. Primary antibodies used for near-infrared immunofluorescence were IBA1 (1:1,000; Wako/Fuji Film 019–19,741; RRID: AB_839504), glial fibrillary acidic protein (GFAP; 1:1,000; Abcam, ab4674; RRID: AB_304558), Synapsin 1 (1:1,000; Cell Signaling Technologies 5297; RRID: AB_2616578), NeuN (1:1,000; Millipore, ABN91; RRID: AB_11205760), myelin basic protein (1:3,000; Antibodies.com, A85321; RRID: AB_2748906), and PSD-95 (1:1,000; Cell Signaling Technologies 3450; RRID: AB_2292883). Following primary antibody incubation, tissue was washed 6 × 10 min in TBS-Tx, followed by incubation for 2 h at room temperature in goat anti-rabbit IRDye 680RD (LiCor 926-68071; RRID: AB_10956166) or donkey anti-chicken IRDye 800CW (LiCor 926-32218; RRID: AB_1850023) secondary antibodies diluted at 1:500 in TBS-Tx containing 2% goat serum. Tissue was washed 6 × 10 min in TBS-Tx, mounted on microscope slides and processed through ethanol and xylenes before cover slipping with Cytoseal-60 (Thermo, 831016). Microscope slides were then imaged on a LiCor Odyssey near-infrared imaging system with ImageStudio software (v5.2.5, LiCor Biosciences) at 21 μm, lowest quality and 0.2 mm offset. Intensities for the 700 and 800 channels were kept constant at 2 for all slides. Following imaging, ImageStudio software was used to draw regions of interest around the hippocampus and cortex. For each animal ~12 tissue sections spanning the rostral-caudal extent of the hippocampus and overlying cortex were analyzed. The signal intensity for the 700 and 800 channels was quantified and normalized to the area of the respective ROIs. The signal intensity normalized to the ROI area was averaged across all sections analyzed within an animal. High magnification images of LiCor immunofluorescent stains were obtained with a Nikon Eclipse 90i fluorescence microscope.

### Western blotting

Dissected hippocampi were homogenized in 10 volumes (w/v) of brain homogenization buffer (50 mM Tris pH 7.4, 274 mM NaCl, 5 mM KCl) containing protease (10 μg/mL pepstatin, leupeptin, bestatin and aprotinin, and 1 mM PMSF) and phosphatase inhibitors (1 mM tetra-sodium pyrophosphate decahydrate, 10 mM beta-glycerophosphate disodium salt pentahydrate, 1 mM sodium orthovanadate, 10 mM sodium fluoride) by sonication using 10 short pulses at power 2 (Misonix XL-200). Crude lysates were centrifuged at 27,000x*g* for 20 min at 4°C and the supernatant (S1) was collected while the pellet (P1) was further processed to obtain sarkosyl insoluble tau samples. P1 was homogenized in 5 volumes of brain pellet homogenization buffer (0.8 M NaCl, 10% sucrose, 10 mM Tris pH 7.4, 1 mM EGTA) and centrifuged at 27,000x*g* for 20 min at 4°C. Sarkosyl was added to the resultant supernatant (S2) to yield a final concentration of 1% sarkosyl and was incubated at 37°C for 1 h. Following incubation, the sarkosyl - containing S2 fraction was centrifuged at 200,000x*g* for 1 h at 4°C. The resultant pellet (P3) was resuspended in brain pellet homogenization buffer and used for analysis as sarkosyl insoluble tau. Protein content was determined using a Pierce rapid gold BCA kit (Thermo, A53225) according to the manufacturer’s instructions. For analysis of tau isoforms, S1 lysate samples were dephosphorylated overnight at 37° C using FastAP Thermosensitive Alkaline Phosphatase (Fermentas, EF0651). For all samples, 20–25 μg of protein was mixed with 6x Laemmli sample buffer and heated to 98° C for 5 min. Proteins were separated by SDS-PAGE using AnyKD Criterion TGX gels (BioRad, 5671124) or 10% Criterion TGX gels (5671034) and transferred to BioTrace nitrocellulose membranes (VWR, 27376-991). Membranes were blocked in 2% non-fat dry milk in TBS prior to incubation overnight at 4° C in primary antibodies. Primary antibodies used were IBA1 (1:500; Fuji Film 016-20,001; RRID: AB_839504), GFAP (1:5000; Abcam, ab4674; RRID: AB_304558), Synapsin 1 (1:1,000; Cell Signaling Technologies 5297; RRID: AB_2616578), NeuN (1:500; Millipore, ABN91; RRID: AB_11205760), myelin basic protein (1:1,000; Abcam, ab123499; RRID: AB_2748906), PSD-95 (1:1,000; Cell Signaling Technologies 3450; RRID: AB_2292883), PHF1 (1:50,000; Binder/Kanaan Lab; RRID: AB_2315150), AT8 (1:10,000; Thermo MN102; RRID: AB_223647), pS422 (1:2,000; Abcam 79,415; RRID: AB_1603345), Tau5 (1:500,000; Kanaan Lab; RRID: AB_2721194), 3R tau (1:1,000; Millipore 05–803; RRID: AB_310013), 4R Tau (1:2,000; Cosmobio United Stetes, TIP-4RT-P01; RRID: AB_2814647), Beta III tubulin (1:10,000)(Caccamo et al., 1989), MAP2 (1:1,000; Cell Signaling Technologies 8707; RRID: AB_2722660), and GAPDH (1:2,000; Cell signaling Technologies 5174; RRID: AB_10622025). Membranes were then washed 3 × 5 min in TBS containing 0.1% tween 20 (TBS-T) followed by incubation for 2 h at room temperature in either goat anti-mouse IRDye 800CW (LiCor 926-32210; RRID: AB_621842), goat anti-rabbit IRDye 680RD (LiCor 926-68071; RRID: AB_2313606), or donkey anti-chicken IRDye 800CW (LiCor 926–32218; RRID: AB_1850023) diluted at 1:20,000. Membranes were washed 3 × 5 min in TBS-T and then imaged on a LiCor Odyssey near-infrared imaging system with ImageStudio software (v5.2.5, LiCor Biosciences).

### Statistical analysis

Statistical analysis of data and graphing of results were performed using GraphPad Prism. For all end points other than the radial arm water maze, data was compiled, and multiple unpaired *t*-tests corrected for multiple comparisons using the Holm-Sidak method were run to compare sexes within a given age and genotype. If there was no significant difference between sexes within a given genotype and age the sexes were combined. After which a two-way ANOVA with a *post-hoc* Tukey test was used to determine differences between groups. For the radial arm water maze, all data was compiled and analyzed as a multifactorial repeated measures ANOVA with a *post-hoc* Tukey test for multiple comparisons. If there were no statistical differences between sexes within an age/genotype, sexes were combined and analyzed as a two-way repeated measures ANOVA with a post-hoc Tukey test for multiple comparisons. Statistical data for all analyses that were used to determine sex differences are included in Supplementary Tables S1–S3. In all cases significance was set at *p* ≤ 0.05.

### Results

#### Behavioral assessment of MAPT KI mice

MAPT KI mice do not show memory impairments in the Y-maze task at 12-months of age (Saito et al., 2019). Beyond this report, a detailed investigation of the behavioral phenotype of MAPT KI mice across their adult lifespan was missing. Thus, we began our characterization of the MAPT KI mouse using a battery of behavioral tests. Male and female MAPT KI and WT mice at ages 6, 12, 18 and 24 m completed a set of behavioral tests selected to probe locomotor activity, motivation, anxiety - like behavior, object recognition and spatial memory. For all behavioral tests, data analyses did not detect sex differences within a given age and genotype (Supplementary Table S1). Thus, data for all behavioral assays is presented with sexes combined within age and genotype.

First, we performed the open field task as a measure of locomotor behavior and anxiety-like behavior (Seibenhener and Wooten, 2015). As a measure of anxiety-like behavior, we quantified the percent of time mice spent in the center square of the open field apparatus. There were no significant differences in percent of time in the center square between WT and MAPT KI animals at any age analyzed, nor were there significant differences across ages within genotypes (Figures 1A,B). We also quantified total distance traveled within the open field apparatus as a measure of locomotor activity. We found a significant decrease in the total distance traveled in 24 m WT animals compared to 24 m MAPT KI mice and 12 m WT mice. We also observed a significant decrease in total distance traveled in 18 m WT mice compared to 12 m WT (Figures 1A,C). Interestingly, we did not observe age-associated declines in locomotor activity in MAPT KI mice. These data indicate that *MAPT* gene KI does not affect general anxiety-like behavior (as indexed by the open field task,) but does prevent the age-associated decrease in locomotor activity observed in WT animals.

We next performed the object location task (OLT) and novel object recognition task (NORT). This task takes advantage of mice’s natural tendency to explore novelty and can serve as a metric of spatial memory and motivation that does not rely on extrinsic motivators (such as fear or reward)(Denninger et al., 2018). Figure 1D shows a schematic of this task. In trial 1, animals are allowed to freely explore 2 identical objects within an open field apparatus. Supplementary Figure S2 shows that all groups spent an equal proportion of time exploring both objects during training in trial 1, confirming a lack of bias towards either object or the respective sides of the open field apparatus. In trial 2, one of the objects is relocated to another corner, after which animals are allowed to freely explore. Memory for the objects original location is reflected by the amount of time the mice spend investigating the relocated object (RO; Figure 1D). During the OLT, MAPT KI mice and WT mice spent similar time exploring the RO at all ages (Figures 1E,F). Finally in trial 3, the object that was not moved in trial 2 is replaced with a novel object (NO; Figure 1D), and memory for the original object is reflected by the amount of time spent investigating the NO. Here, we found that MAPT KI mice spent greater than 50% of time exploring the NO, while WT animals spent slightly less time exploring the NO. There were no differences in time exploring the NO between any groups (Figures 1G,H). These results indicate the *MAPT* gene KI does not affect spatial memory or object recognition.

As another index of spatial memory, we performed the radial arm water maze (Alamed et al., 2006). In this task, animals are placed in a pool of water separated into arms and are required to find an escape platform located at the end of one of the arms. Animals must use spatial cues to locate the escape platform from different starting arms. On day 1, animals are first trained by alternating between a visible and hidden escape platform (blocks 0–5), while on day 2 (blocks 6–10) the escape platform is always hidden. All animals were able to successfully learn the location of the escape platform by the end of day 1 (block 5), and there were no significant differences in either the number of errors or the latency to find the escape platform between any of the groups (Figures 1I,J). These data provide further evidence that MAPT KI mice have normal spatial memory from 6 to 24 m of age.

Finally, we performed the rotarod task as a metric of locomotor function. Here, animals are placed on a rotating rod that accelerates from 4- to 400 RPMs over a 5 min period. After training, the latency to fall is recorded over 4 trials and averaged. There was an age-associated decrease in the latency to fall in both WT and MAPT KI mice after 6 m of age (Figure 1K). More specifically, the 12, 18 and 24 m mice (MAPT KI and WT) showed a significant decrease in latency to fall from the rod when compared to 6 m mice in both the MAPT KI and WT groups. Further, there was a significant decrease in latency to fall from the rod in 18 m WT mice compared to 12 and 18 m MAPT KI mice, and 12 m WT mice. These results demonstrate an age-associated decrease in locomotor function in all animals, that is, partially delayed in MAPT KI mice.

#### Neuropathological assessment of MAPT KI mice

We next sought to provide a detailed characterization of potential neuropathological changes that may occur due to *MAPT* gene KI. To this end, we used a combination of semi-quantitative immunofluorescence and biochemistry to quantify markers of neurons (NeuN), pre- and post-synapses (synapsin 1 and PSD-95, respectively) as well as glia (GFAP for astrocytes, IBA1 for microglia and MBP for oligodendrocytes) in the hippocampus (Hp) and cortex (Cx) of MAPT KI and WT mice at 6, 12 and 24 m of age. Again, for all analyses we did not detect a significant difference between sexes within a given age and genotype (Supplementary Table S2), thus, sexes were combined within genotypes at each age.

**Figure 2:**
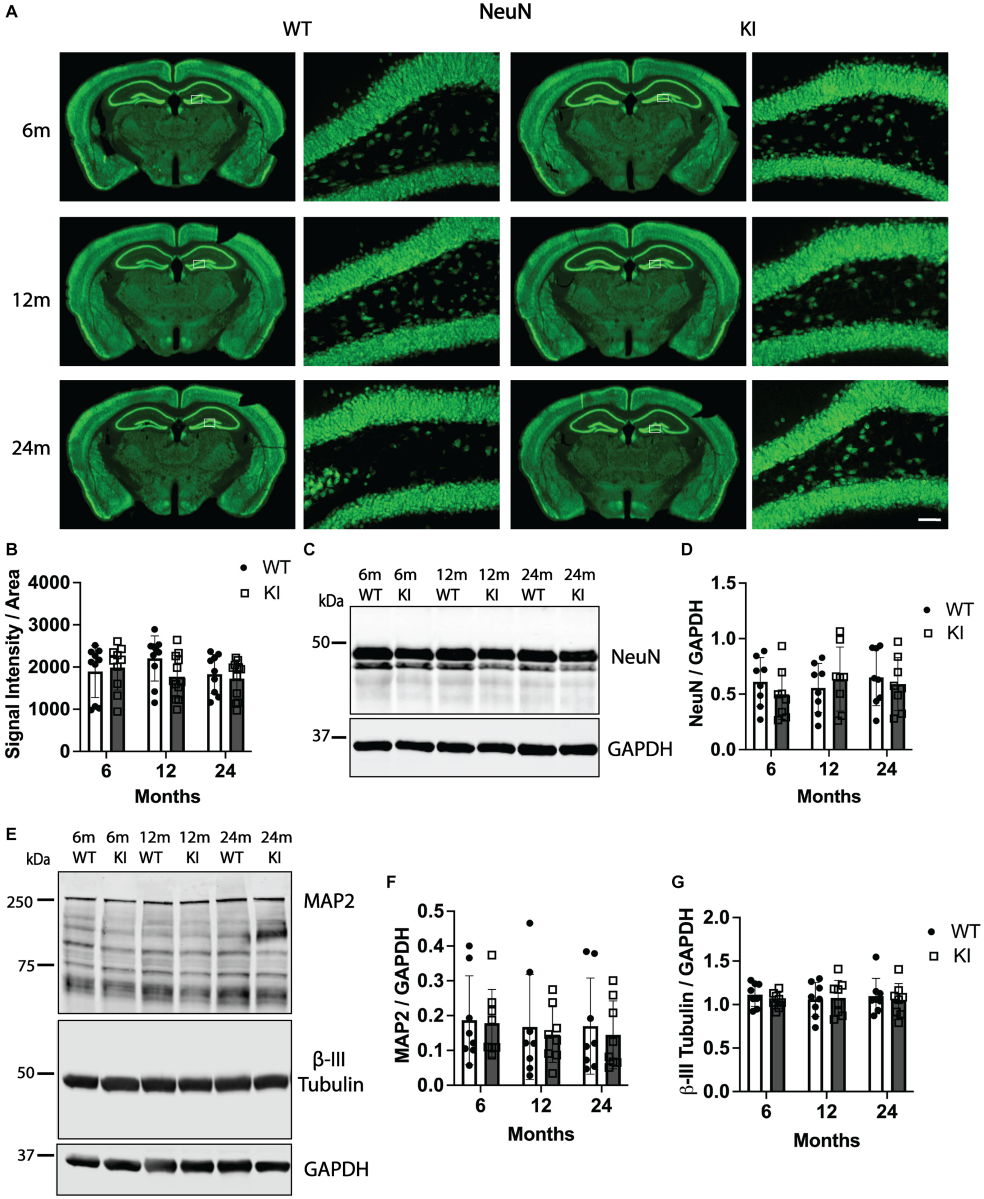
Quantification of neuronal and cytoskeletal markers in the hippocampus of MAPT KI and WT mice. Tissue from 6, 12, and 24 month old male and female human MAPT knock-in (MAPT KI or KI) and wild type (WT) mice (*n* = 4–5/group) was processed for immunofluorescent detection neuronal nuclei (NeuN) in the hippocampus (panels **A,B**), as well as biochemical quantification of NeuN (panels **C,D**), microtubule-associated protein 2 (MAP 2; panels **E,F**) and β-III tubulin (panels **E,G**) from hippocampal lysates. For all measures, no sex differences were detected within a given age and genotype (see Supplementary Tables S2.1A–S2.3A for data on individual sexes). Sexes were combined within age and genotype for all subsequent analyses. **(A)** Representative images of NeuN immunofluorescence in MAPT KI and WT mice. High magnification images of NeuN immunofluorescence correspond to the area within the white box in the low magnification images. **(B)** Quantification of NeuN fluorescence intensity normalized to the area of the respective hippocampal sections. **(C)** Representative immunoblots of NeuN and glyceraldehyde phosphate dehydrogenase (GAPDH) from hippocampal lysates. **(D)** Quantification of NeuN normalized to GAPDH in hippocampal lysates. **(E)** Representative immunoblots of MAP2, β-III tubulin and GAPDH from hippocampal lysates. **(F)** Quantification of MAP2 A/B (~250 kDa band) normalized to GAPDH. **(G)** Quantification of β-III tubulin normalized to GAPDH. For all histograms, individual data points are shown while bars represent the mean of groups ± standard deviation (*n* = 8–10/group for combined sexes). Scale bar in the bottom right panel of **(A)** is 50 μm and applies to all high magnification images.

Figure 2 shows quantification of NeuN in the Hp. Quantification of NeuN signal intensity in the Hp did not reveal any significant differences between genotypes or across ages within genotypes. Representative low magnification images of NeuN immunoreactivity in coronal tissue sections encompassing the ventral Hp and overlying cortex as well as high magnification images of NeuN immunofluorescence in the granule cell layer of the dentate gyrus are shown in Figure 2A. We also quantified NeuN immunofluorescence intensity in the Cx and did not detect any significant differences between any groups (Supplementary Figure S3). To corroborate these results, we quantified NeuN by SDS-PAGE and western blotting from Hp lysates. Again, we did not detect any significant differences in Hp NeuN protein levels between groups (Figures 2C,D). Finally, we also quantified levels of two additional proteins, one that has a functional interaction with tau (β-III tubulin) and another that is a different neuronal microtubule-associated protein (MAP2). There were no changes in levels of β-III tubulin or MAP2 in the Hp of MAPT KI mice compared to WT mice at any age, or across ages within either genotype (Figures 2E–G).

**Figure 3:**
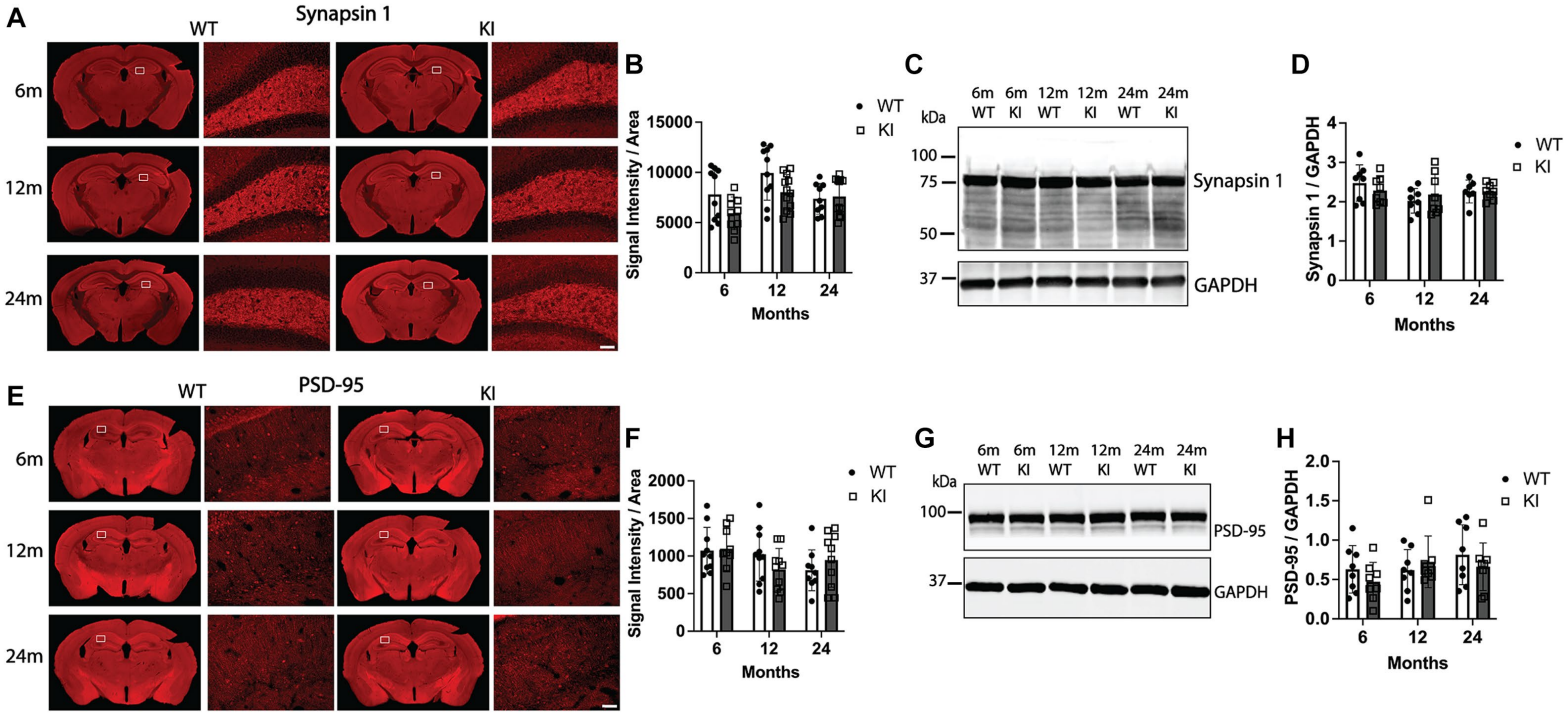
Quantification of synaptic markers in the hippocampus of MAPT KI and WT mice. Tissue from 6, 12, and 24 months old male and female human MAPT knock-in (MAPT KI or KI) and wild type (WT) mice (*n* = 4–5/group) was processed for immunofluorescence detection of the presynaptic marker, synapsin 1 **(A,B)**, and the post-synaptic marker, post synaptic density 95 (PSD-95; **E,F**) in the hippocampus, as well as biochemical quantification of synapsin 1 **(C,D)** and PSD-95 **(G,H)** from hippocampal lysates. For all endpoints, no sex differences were detected within a given age and genotype (see Supplementary Tables S2.4A–S2.5B for data on individual sexes). Sexes were combined within age and genotype for all subsequent analyses. **(A)** Representative images of synapsin 1 immunofluorescence in MAPT KI and WT mice. High magnification images of synapsin 1 immunofluorescence correspond to the area within in the box in the low magnification images. **(B)** Quantification of synapsin 1 fluorescence intensity normalized to the area of the respective hippocampal sections. **(C)** Representative immunoblots of synapsin 1 and glyceraldehyde phosphate dehydrogenase (GAPDH) from hippocampal lysates. **(D)** Quantification of synapsin 1 normalized to GAPDH in hippocampal lysates. **(E)** Representative images of PSD-95 immunofluorescence in MAPT KI and WT mice. High magnification images correspond to the area within in the box in the low magnification images. **(F)** Quantification of PSD-95 fluorescence intensity normalized to the area of the respective hippocampal sections. **(G)** Representative immunoblots of PSD-95 and GAPDH from hippocampal lysates. **(H)** Quantification of PSD-95 normalized to GAPDH in hippocampal lysates. For all histograms, individual data points are shown while bars represent the mean of groups ± standard deviation (*n* = 8–10/group for combined sexes). Scale bar in the bottom right panel of **(A,E)** is 50 μm and applies to all high magnification images.

To evaluate possible degeneration of pre- and post-synapses, we quantified levels of synapsin 1 (pre-synaptic marker) and PSD-95 (post-synaptic marker) in the Hp and Cx. Figures 3A,B shows quantification of synapsin 1 immunofluorescence intensity in the Hp. There was no change in synapsin 1 intensity within the Hp (Figure 3B) or the Cx (Supplementary Figures S4A,B) between any groups. Quantification of synapsin 1 by western blotting from Hp lysates did not detect significant differences between genotypes or across age within genotype (Figures 3C,D), confirming the synapsin 1 immunofluorescence analysis. We next performed an identical analysis to quantify levels of PSD-95 in the Hp and Cx by immunofluorescence analysis. There were no significant differences in PSD-95 immunofluorescence intensity between WT and MAPT KI animals at any age, nor within genotypes across ages in the Hp (Figures 3E,F) or in the Cx (Supplementary Figures S4C,D). Similarly, quantification of PSD-95 protein in Hp lysates by western blot did not detect differences between any groups analyzed (Figures 3G,H). Taken together, these results suggest MAPT KI animals do not have significant alterations in synaptic integrity.

**Figure 4:**
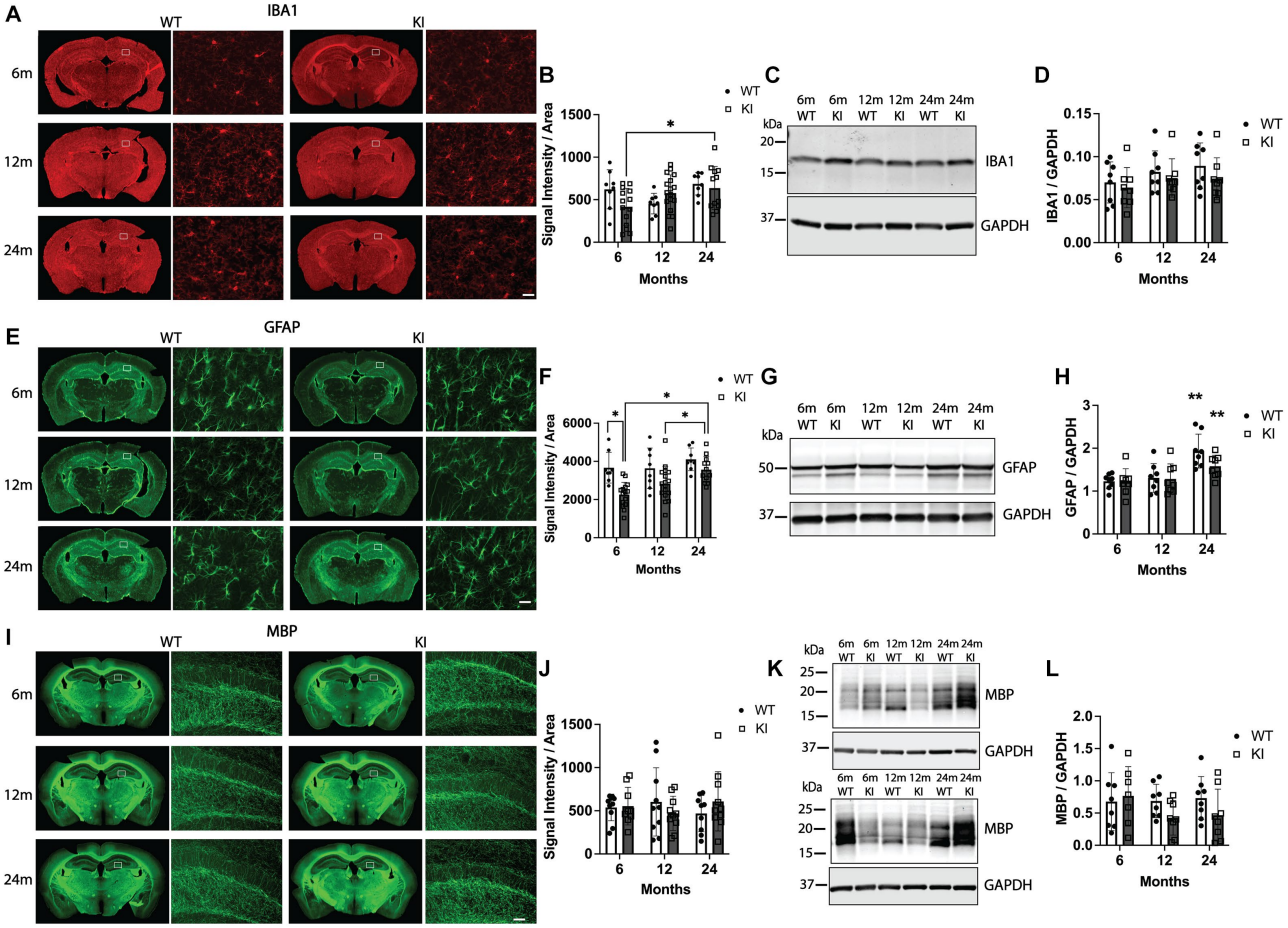
Quantification of glial markers in the hippocampus of MAPT KI and WT mice. Tissue from 6, 12, and 24 month (m) old male and female human MAPT knock-in (MAPT KI or KI) and wild type (WT) mice (*n* = 4–5/group) was processed to perform immunofluorescence detection of the microglial marker, ionized calcium adapter molecule 1 (IBA1; **A,B**), the astrocytic marker glial fibrillary acidic protein (GFAP; **E,F**) and the oligodendrocyte marker, myelin basic protein (MBP; **I,J**) in the hippocampus. Fresh hippocampal tissue was processed for biochemical quantification of IBA1 **(C,D)**, GFAP **(G,H)**, and MBP **(K,L)**. For all endpoints, no sex differences were detected within a given age and genotype (see Supplementary Tables S2.6A–S2.8B for data on individual sexes). Sexes were combined within age and genotype for all subsequent analyses. **(A)** Representative images of IBA1 immunofluorescence in MAPT KI and WT mice. High magnification images of IBA1 immunofluorescence correspond to the area within the box in the low magnification images. **(B)** Quantification of IBA1 fluorescence intensity normalized to the area of the respective hippocampal sections. **(C)** Representative immunoblots of IBA1 and glyceraldehyde phosphate dehydrogenase (GAPDH) from hippocampal lysates. **(D)** Quantification of IBA1 normalized to GAPDH in hippocampal lysates. **(E)** Representative images of GFAP immunofluorescence in MAPT KI and WT mice. High magnification images of GFAP immunofluorescence correspond to the area within the box in the low magnification images. **(F)** Quantification of GFAP fluorescence intensity normalized to the area of the respective hippocampal sections. **(G)** Representative immunoblots of GFAP and GAPDH from hippocampal lysates. **(H)** Quantification of GFAP normalized to GAPDH in hippocampal lysates. There was a significant increase in hippocampal GFAP protein levels in both WT and MAPT KI mice at 24 m of age compared to 6 m and 12 m old mice. **(I)** Representative images of MBP immunofluorescence in MAPT KI and WT mice. High magnification images of MBP immunofluorescence correspond to the area within the box in the low magnification images. **(J)** Quantification of MBP immunofluorescence intensity normalized to the area of the respective hippocampal sections. **(K)** Representative immunoblots of MBP and GAPDH from hippocampal lysates. **(L)** Quantification of MBP normalized to GAPDH in hippocampal lysates. For all histograms, individual data points are shown while bars represent the mean of groups ± standard deviation. * *p* ≤ 0.05. ** Significantly different than 6 m and 12 m ages (both WT and MAPT KI; *p* ≤ 0.05). For all comparisons *n* = 8–10/group for combined sexes. Scale bars in the lower left panel of **(A,E)** are 25 μm and apply to all high magnification images in **(A,E)**. Scale bar in lower left panel of **(I)** is 50 μm and applies to all high magnification images in **(I)**.

We next performed the same combination of immunofluorescence quantification combined with biochemical quantification of glial cell markers. Figures 4A,B shows analysis of IBA1 (a pan-microglia marker) immunofluorescence. We detected a significant increase in IBA1 fluorescence intensity in the Hp in 24 m MAPT KI mice compared to 6 m MAPT KI (Figures 4A,B). No other changes were observed in IBA1 fluorescence intensity in the Hp or the Cx (Supplementary Figures S5A,B). Quantification of IBA1 protein in the Hp by western blot detected no differences between any groups (Figures 4C,D). We next performed the same analysis for the astrocytic marker GFAP. Figures 4E,F shows quantification of GFAP immunofluorescence in the Hp. Within genotypes, there was a significant increase in GFAP fluorescence intensity in the Hp of 24 m MAPT KI mice compared to 6 and 12 m MAPT KI mice. In contrast, in WT mice GFAP fluorescence intensity did not change with age. Within age groups, the only difference between WT and MAPT KI mice was that 6 m WT animals showed significantly greater Hp GFAP fluorescence intensity (Figure 4F). We observed similar results in the Cx, with a significant increase in GFAP fluorescence intensity in 24 m MAPT KI mice compared to 6 m MAPT KI (Supplementary Figures S5C,D). When we quantified levels of GFAP protein in Hp lysates by western blot, we detected a significant increase in GFAP protein in 24 m mice (WT and MAPT KI) compared to 6 and 12 m mice (Figures 4G,H). Finally, quantification of the oligodendrocyte-associated protein, MBP, is shown in Figures 4I–L (Hp) and Supplementary Figures S5E,F (Cx). There were no significant differences in MBP fluorescence intensity between groups in the Hp or the Cx. When Hp MBP was quantified by western blot no differences were detected, however, there was variability between animals that was consistent within all groups (sex, genotype, and age) (Figures 4K,L). Additional representative MBP blots are shown to demonstrate this variability (Figure 4K). Taken together, these results suggest that KI of the *MAPT* gene does not alter glial numbers or glial activation compared to WT animals, and that both WT and MAPT KI mice show an age-associated increase in GFAP levels.

#### Assessment of tau protein levels, isoform expression, phosphorylation, and conformation in MAPT KI mice

**Figure 5:**
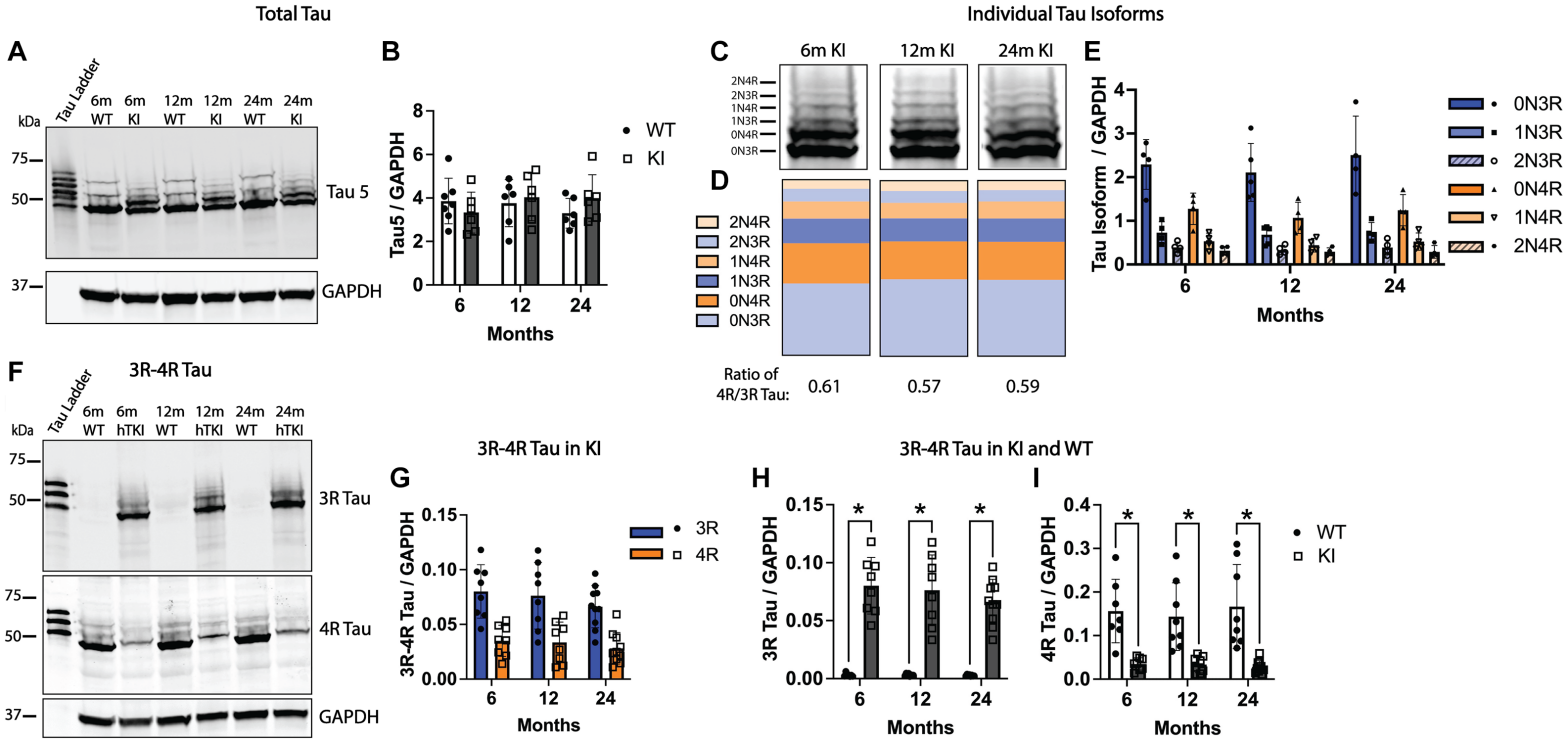
Quantification of tau in MAPT KI and WT mice. Tissue from 6, 12 and 24-month old male and female human MAPT knock-in (MAPT KI or KI) and wild type (WT) (*n* = 2–5/group) was processed for biochemical detection of total tau **(A,B)**, individual tau isoforms **(C–E)**, and 3R/4R tau **(F–I)**. For all endpoints, no sex differences were detected within a given age and genotype (see Supplementary Tables S3.1A–S3.3A for data on individual sexes). Sexes were combined within age and genotype for all subsequent analyses. **(A)** Representative immunoblots using the pan-tau antibody, Tau5, and glyceraldehyde phosphate dehydrogenase (GAPDH). **(B)** Quantification of Tau5 normalized to GAPDH. **(C)** Representative immunoblots showing individual tau isoforms. **(D)** Quantification of the relative proportion of individual tau isoforms to the total amount of tau. **(E)** Quantification of individual tau isoforms normalized to GAPDH. **(F)** Representative immunoblot showing 3R tau, 4R tau and GAPDH. **(G)** Quantification of 3R and 4R tau normalized to GAPDH in MAPT KI mice. **(H)** Quantification of 3R tau normalized to GAPDH. **(I)** Quantification of 4R tau normalized to GAPDH. For all histograms, individual data points are shown while bars represent the mean of groups ± standard deviation. * *p* ≤ 0.05 (*n* = 4–9/group for combined sexes).

We quantified total tau protein and individual tau isoforms in the Hp across the adult lifetime of MAPT KI mice. To quantify total tau, we performed western blotting using the pan-tau antibody, Tau 5, using Hp S1 lysates from MAPT KI and WT animals at 6, 12 and 24 m of age. Figures 5A,B shows similar total tau levels between MAPT KI and WT mice. WT mice solely expressed 4R tau while MAPT KI mice express all 6 isoforms of human tau. Further, Hp total tau levels are not altered with age in the MAPT KI mouse. We next quantified levels of individual tau isoforms in MAPT KI mice at 6, 12, and 24 m of age (Figures 5C–E). There was no change in the relative abundance of the individual tau isoforms over the adult lifetime of the MAPT KI mice. In general, there was an inverse relationship between the length of the tau isoforms and the relative abundance. The most abundant tau isoforms in the Hp of MAPT KI mice were the 0 N isoforms, followed by 1 N and lastly the 2 N isoforms being the least abundant. Within these isoforms 3R tau was more abundant than the corresponding 4R isoform (e.g., 0N3R > 0N4R; Figures 5C–E). The ratio of 4R/3R tau did not alter with age in MAPT KI mice and was 0.61 ± 0.01 at 6 m, 0.57 ± 0.02 at 12 m, and 0.59 ± 0.02 at 24 m, which is in correspondence with previous reports (Saito et al., 2019). To corroborate these findings, we next quantified levels of 3R and 4R tau in the Hp using antibodies specific to 3R and 4R tau. Again, we did not detect any change in levels of 3R or 4R tau in the Hp of MAPT KI mice across age (Figures 5F,G). Figures 5H,I shows a comparison of 3R and 4R tau between MAPT KI and WT animals at 6, 12, and 24 m of age. WT and MAPT KI mice have roughly equal total tau protein expression in the HP (Figures 5A,B) despite WT animals not expressing 3R tau (Figures 5F,H). Accordingly, WT mice express more 4R tau than MAPT KI animals (Figures 5F,I). Taken together, these data show MAPT KI mice produce physiological levels of tau, and tau isoform levels remain constant with age.

**Figure 6:**
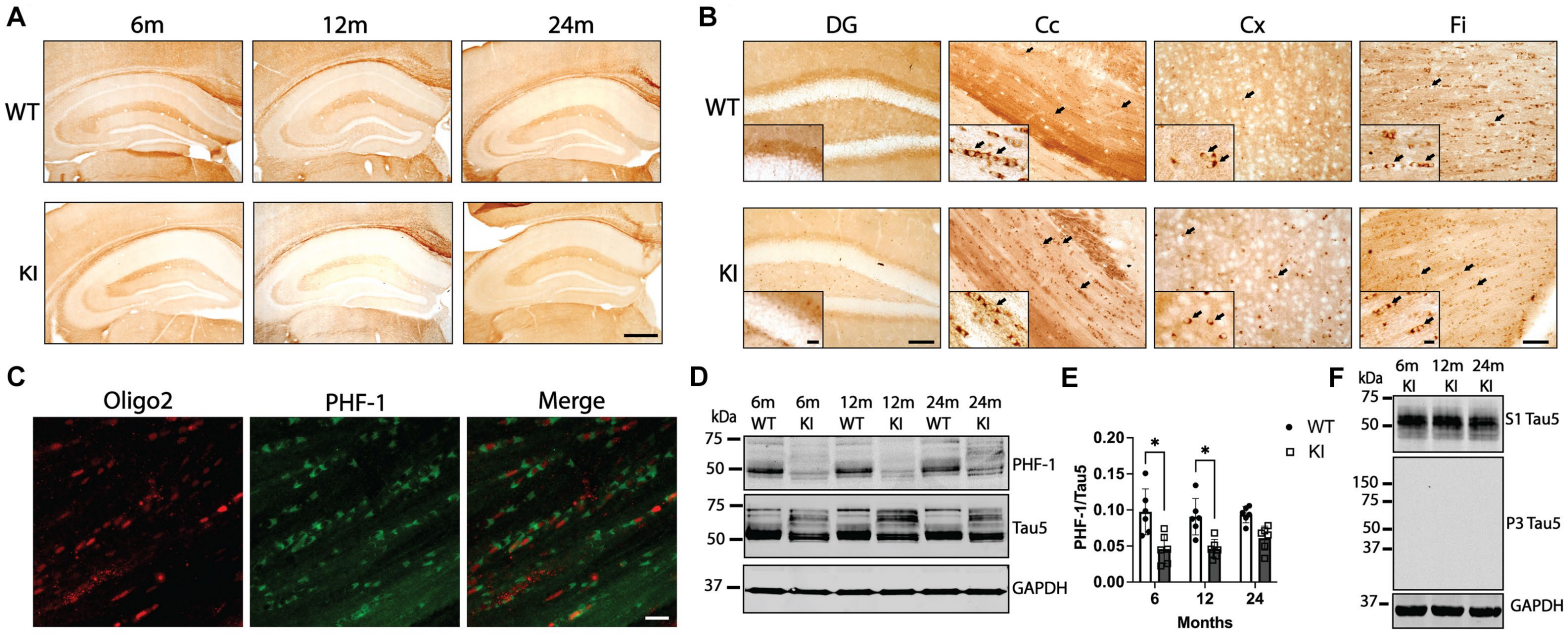
PHF1 tau phosphorylation in MAPT KI and WT mice. Tissue from 6, 12 and 24-month old male and female human MAPT knock-in (MAPT KI or KI) and wild type (WT) mice (*n* = 4–5/group) was processed for immunohistochemical detection of PHF1 (phosphorylated Ser396/Ser404; **A,B**) or immunofluorescence detection of PHF1 and the oligodendrocyte marker oligo2 **(C)**. **(A)** Representative images of PHF1 immunoreactivity in MAPT KI and WT animals. **(B)** Representative high magnification images of PHF1 immunoreactivity in the dentate gyrus (DG), corpus callosum (Cc), cerebral cortex (Cx) and fimbria (FI). Arrows indicate PHF1+ cells that morphologically resemble oligodendrocytes. Insets in **(B)** show high magnification images of PHF1 immunoreactivity in the respective brain regions. **(C)** Immunofluorescence detection of Oligo2 and PHF1 in the Cc of KI mice show PHF1+ oligodendrocytes. **(D)** Fresh hippocampal tissue was processed for biochemical quantification of PHF1 tau (*n* = 4/group). For all biochemical analyses, no sex differences were detected within a given age and genotype (see Supplementary Table S3.4A). Sexes were combined within age and genotype for all subsequent comparisons. **(D)** Representative immunoblots of hippocampal PHF1, Tau5 and glyceraldehyde phosphate dehydrogenase (GAPDH). **(E)** Quantification of PHF1 tau normalized to Tau5. **(F)** Representative immunoblots showing sarkosyl soluble tau (S1), sarkosyl insoluble tau (P3) and GAPDH. For all histograms, individual data points are shown while bars represent the mean of groups ± standard deviation. *p ≤ 0.05 (*n* = 6–8/group for combined sexes). Sale bar in the lower right panel of **(A)** is 500 μm and applies to all panels in **(A)**. Scale bar in lower DG panel of **(B)** is 100 μm and applies to upper DG panel in **(B)**. Scale bar in the inset of the lower DG panel in **(B)** is 25 μm and applies to inset in the upper DG panel in **(B)**. Scale bar in lower Fi panel of **(B)** is 50 μm and applies all Cc, Cx and Fi panels in **(B)**. Scale bar in the inset of the lower Fi panel in **(B)** is 10 μm and applies to the insets in all Cc, Cx and Fi panels in **(B)**. Scale bar is lower right panel of **(C)** is 10 μm and applies to all panels in **(C)**.

Finally, we investigated tau phosphorylation and abnormal tau conformations in MAPT KI and WT mice at 6, 12, and 24 m of age. To determine tau phosphorylation status, we performed IHC and western blotting for AT8 (phosphorylation within aa198-210) (Biernat et al., 1992; Goedert et al., 1995; Malia et al., 2016), pS422 (phosphorylation at Ser 422) (Kimura et al., 1996; Guillozet-Bongaarts et al., 2006), and PHF1 (phosphorylation at Ser 396/ Ser 404) (Greenberg et al., 1992). We did not detect any AT8 or pS422 signal by either western blot or IHC in MAPT KI or WT animals of any age (Supplementary Figure S6). In contrast, there was significant PHF1 immunoreactivity in both MAPT KI and WT animals (Figure 6). PHF1 IHC produced a combination of diffuse parenchymal labeling [typical neuronal tau staining pattern (Kanaan and Grabinski, 2021)] as well as small intensely stained somata found throughout the parenchyma and particularly enriched in white matter (arrows in Figure 6B). Based on prior work describing the normal distribution of tau in the CNS (Migheli et al., 1988; Lopresti, 2002; Kubo et al., 2019; Kanaan and Grabinski, 2021), as well as the location and morphology of these cells, we sought to confirm these cells were oligodendrocytes. We performed dual immunofluorescence for PHF1 and the oligodendrocyte marker Oligo2. Figure 6C shows that the PHF1+ cells in the white matter of the corpus callosum also label with Oligo2, confirming PHF1+ oligodendrocytes in white matter of both MAPT KI and WT mice. PHF1+ cells in the parenchyma also labeled with Oligo2 (data not shown). We quantified PHF1 tau in the Hp by western blot (Figures 6D,E). There was significantly more PHF1 signal in the Hp of WT animals compared to MAPT KI animals at 6 and 12 m of age. Levels of PHF1 within genotype did not significantly change across ages, despite a non-significant increase in 24 m MAPT KI mice. We next performed IHC using antibodies that recognize oligomeric tau (TOC1)(Patterson et al., 2011), and abnormally folded tau (TNT2, exposure of N-terminal domain of tau)(Combs et al., 2016). We did not detect any appreciable immunoreactivity using either TOC1 or TNT2 antibodies (data not shown). Finally, we analyzed levels of tau in the sarkosyl insoluble P3 fraction to determine if insoluble tau accumulates with advancing age in MAPT KI mice. Figure 6F shows the expected presence of soluble tau in the S1 fraction and a lack of tau in the insoluble P3 fraction. These data show that tau in MAPT KI mice does not exhibit abnormal conformations or form insoluble species. Tau is robustly phosphorylated at the PHF1 site in neurons and oligodendrocytes under physiological conditions in MAPT KI mice, but not at sites more closely associated with disease in humans (i.e., AT8 and pS422).

## Discussion

Tau is a functionally diverse protein that plays important roles in normal neurophysiology and human disease pathogenesis. Experimental research models are necessary to accurately dissect the *in vivo* function and dysfunction of tau protein. The MAPT KI mouse expresses all 6 isoforms of human tau protein at physiological levels without the inherent drawbacks of transgenesis or overexpression. We believe this makes MAPT KI mice one of the best available animal models to study tau function *in vivo*. Due to the utility of this model, an in-depth characterization of the effects of *MAPT* gene KI was warranted. To this end, we have completed a battery of behavioral and neuropathological assays from 6 m to 24 m of age in MAPT KI mice and compared their performance to C57BL/6 J WT mice. Finally, we provided a detailed analysis of total tau expression, the relative expression of tau isoforms, as well as investigations into tau phosphorylation and abnormal tau conformations in MAPT KI mice. Taken together, we have confirmed and extended previous reports showing that KI of the human *MAPT* gene does not cause overt pathology or behavioral abnormalities.

We performed a detailed behavioral analysis that revealed MAPT KI mice show normal cognitive and locomotor behavior at all ages examined. In fact, MAPT KI mice display a resilience to the age-associated decreases in locomotor function that occurs in WT animals. More specifically, 24 m WT mice showed a significant decrease in locomotor activity in the open field task that was not observed in MAPT KI mice. In the rotarod test, MAPT KI mice had a significantly greater latency to fall from the bar at 18 m compared to WT animals, although this difference was abolished by 24 m of age. Several reports have previously linked alterations in murine tau expression to changes in locomotor activity. For instance, hTau mice exhibit increases in total distance traveled in the open field task (Geiszler et al., 2016; Cho et al., 2021). Several tau transgenic mice also show increased locomotor behavior. Young adult PS19 mice (1 m–4 m of age) show increased locomotor activity in the open field task as well as increased swim speed in the Morris water maze (Scattoni et al., 2010), while adult rTg4510 mice (4 m–6 m of age) show a hyperactive locomotor profile (Jul et al., 2015). In a similar fashion germline tau KO mice show an increase in total distance traveled in the open field task (Ikegami et al., 2000).

Conversely, shRNA mediated knockdown of endogenous murine tau causes impaired performance on the rotarod task (Velazquez et al., 2018). Several tau transgenic mice also show impaired motor performance late in life, including some lines that show locomotor hyperactivity early in life. For instance, aged PS19 mice (9–10 m of age) show limb weakness progressing to paralysis (Yoshiyama et al., 2007), while aged JNPL3 mice (10 m of age) develop severe limb weakness and dystonic posturing (Lewis et al., 2000). Unlike tau transgenics, *MAPT* gene KI seems to merely preserve a normal locomotor phenotype throughout life, as opposed to increasing or decreasing locomotion. Published results and those presented herein suggest manipulation of tau can impact motor function, however, the underlying mechanisms are only partially understood. Motor impairment in most tau transgenic mice is an end stage phenotype resulting from degeneration of motor neurons following accumulation of pathological tau in the spinal cord (Lewis et al., 2000; Yoshiyama et al., 2007). In contrast, mechanisms underlying tau-associated increases in locomotor activity are less defined. It is possible that the presence of human tau or loss of murine tau may modulate locomotor activity in rodents. Alternatively, these effects may be caused by neurodevelopmental compensations following germline manipulation of tau. This is an interesting and largely overlooked function of the tau protein and future studies are needed to resolve these findings and shed light onto this potential function of tau.

Our neuropathological assessment did not reveal any remarkable changes in markers of neuronal integrity. Specifically, indices of pre-synapses (synapsin 1), post-synapses (PSD-95), and neuronal cell bodies (NeuN) were unaltered compared to age matched WT animals and did not change with age in the Hp or Cx of MAPT KI mice. This agrees with previous reports that did not detect morphological changes in hippocampal or cortical thickness as measured by MRI in MAPT KI mice (Hashimoto et al., 2019), and supports the general conclusion that *MAPT* gene KI does not cause alterations in neuronal integrity. This also is in line with the lack of behavioral impairment. Alterations in levels of PSD-95 in the Cx of aged rats correlates with impairment in the Morris water maze (Preissmann et al., 2012). Similarly, synapsin 1 KO mice show impairment in the Morris water maze (Corradi et al., 2008). Thus, the lack of neuropathology in the current work corroborates the preserved neurobehavioral function in MAPT KI mice.

Analysis of glial markers revealed an age-associated increase in IBA1 in the Hp of MAPT KI mice, as well as an age-associated increase in Hp GFAP in both MAPT KI and WT mice. These results indicate a proliferation and/or activation of both microglia and astrocytes in the aged MAPT KI brain when compared to young MAPT KI mice. However, an important caveat to this conclusion is that, based on immunofluorescence analysis, GFAP levels were decreased in the Hp of MAPT KI mice compared to WT mice at 6 m of age, after which the increase in Hp GFAP in 24 m MAPT KI mice equalized levels of Hp GFAP between WT and MAPT KI mice. Furthermore, biochemical quantification of Hp GFAP revealed increased levels in 24 m MAPT KI and WT mice compared to 6- and 12 m MAPT KI mice, with no difference between genotypes at 24 m of age. An age-related increase in GFAP is a well-documented phenomenon. For instance, previous reports found increased GFAP mRNA in the Hp of 24 m rats compared to 6–7 m rats (Nichols et al., 1993). This result is directly in line with the observations from the current study. Similarly, GFAP mRNA is increased in the Hp and frontal Cx of aged humans (Nichols et al., 1993), and PET imaging reveals an age- and region-dependent increase in reactive astrocytes in the human brain (Mohamed et al., 2022). Data regarding age-associated increases in microglia are not as consistent. Some studies reveal increased IBA1 immunoreactivity and microglia density in the Hp of aged C57BL/6 mice (20 m–27 m) compared to young (2–3 m) C57BL/6 mice (Mouton et al., 2002). However, other reports in rodents and humans do not find increased microglial density or activation state with age (reviewed in Edler et al., 2021). In the current study, IBA1 was significantly increased only in 24 m MAPT KI mice when quantified by immunofluorescence analysis, not immunoblotting. The reason for the discrepancy between our histological versus biochemical quantification of IBA1 is unclear. The differences may be due to differences in fixed versus fresh tissue processing. Taken together, the increased IBA1 and GFAP levels combined with the lack of overt neuropathology accurately reflect changes observed with normal aging, further supporting the utility of this model to study the human tau protein in the context of aging.

An interesting finding from the current work was the relatively high level of Ser396/S404 (PHF1) phosphorylated tau in both MAPT KI and WT mice. Previous reports revealed low levels of AT8 and pS422 tau, in addition to PHF1 tau, in MAPT KI mice (Saito et al., 2019). However, we did not detect AT8 or pS422 phosphorylated tau species in the current work. Diffuse parenchymal PHF1 immunoreactivity resembling normal tau distribution/reactivity was observed throughout the brain in both genotypes. Additionally, PHF1 reactivity appeared to highlight some axon-enriched regions (e.g., stratum lucidum and stratum lacunosum-moleculare) despite the ubiquitous presence of tau protein throughout the somatodendritic and axonal compartments of neurons (Papasozomenos and Binder, 1987; Kanaan and Grabinski, 2021), suggesting PHF1 phosphorylation preferentially occurs in axons under physiological conditions. Finally, levels of PHF1 positive tau were increased in WT animals compared to MAPT KI animals. The PHF1 antibody was originally developed to immunolabel tau protein in paired helical filaments (Greenberg et al., 1992; Otvos et al., 1994). Since its original description, PHF1 reactivity is commonly used as an index of neurofibrillary tangle pathology in human disease. However, tau function is regulated through post-translational modification, including phosphorylation (Wang and Mandelkow, 2016). Indeed, multiple lines of evidence demonstrate that Ser396/404 phosphorylaion is likely one of many mechanisms that can regulate tau function. For instance, induction of hippocampal long-term depression (LTD) stimulates Ser396/404 phosphorylation, and phosphorylation of tau at Ser396 is necessary for LTD in WT mice (Regan et al., 2015). hTau mice have Ser396/404 phosphorylated tau in the brain starting at a young age, and unlike other phospho-tau species examined, levels of PHF1 do not change with advancing age nor do they correlate with neuropathological changes (Andorfer et al., 2003). Healthy WT mice show an age-related accumulation of Ser396/404 phosphorylated tau in the Hp where PHF1 positive tau is present in synaptic mitochondria (Torres et al., 2021). Finally, WT rats have stable levels of PHF1 positive tau throughout adulthood (Kneynsberg and Kanaan, 2017). These results agree with those presented here. Beginning at the earliest age examined (6 m), we observed Ser396/404 phosphorylated tau in the Hp of both MAPT KI and WT mice, and levels did not significantly change with age in either genotype. Oligodendrocytes normally express relatively robust levels of tau under physiological conditions (Migheli et al., 1988; Lopresti, 2002; Kubo et al., 2019; Kanaan and Grabinski, 2021), and we found high levels of Ser396/404 phosphorylated tau present in oligodendrocytes in both MAPT KI and WT mice. These results support the idea that phosphorylation at Ser396/404 is a normal regulatory mechanism in non-pathological states in both neurons and oligodendrocytes. Future studies are needed to further define the physiological roles of phosphorylated ser396/404 tau in different cell types and cellular compartments.

Prior to the generation of the MAPT KI mouse, the primary mouse model available that expressed all six human tau isoforms was the hTau transgenic mouse. This mouse expresses a human tau transgene from a PAC on a mouse tau knockout background (Andorfer et al., 2003, 2005). hTau mice overexpress all 6 tau isoforms, resulting in tauopathy from a relatively young age. Beginning as early as 2 m of age, hTau mice show accumulation of phosphorylated tau protein in the Hp and Cx. With increasing age, phosphorylated tau accumulates and ultimately deposits in mature, insoluble NFT-like aggregates in the Hp and Cx by 9 m. The deposition of insoluble tau results in functional and structural neuronal abnormalities, significant neurodegeneration, and cognitive impairment around 6 m–9 m of age (Polydoro et al., 2009; Phillips et al., 2011). In contrast, MAPT KI mice do not show overt behavioral or neuropathological abnormalities, including the lack of some disease-associated phosphorylation changes, abnormal conformations, or the formation of insoluble tau aggregates. Differences between these two models suggest that human tau is not inherently neurotoxic, but that excessive tau levels may be problematic. Indeed, most humans live a lifetime without developing tauopathy. It is reasonable to conclude that initiating stimuli are necessary to induce tauopathy, after which the process likely proceeds in a feedforward manner. Collectively, our work and prior studies (Hashimoto et al., 2019; Saito et al., 2019), suggest MAPT KI mice are a model organism that expresses human tau without obligate tau pathology.

Although MAPT KI mice do not spontaneously develop tau-associated pathology, they are still a useful model to investigate mechanisms of tauopathy. As mentioned, tau aggregates in AD are composed of 3R and 4R tau isoforms. Accordingly, aggregates composed of endogenous tau in MAPT KI mice could more closely resemble neurofibrillary tangles than aggregates composed of murine tau or the protein product of a human tau transgene (which usually encode a single tau isoform containing a mutation). Recently, tau seeding models have emerged as an alternative to transgenic or viral vector-based overexpression models of tauopathy. In these models, “seeds” of misfolded tau are injected into the rodent brain. The tau seeds are subsequently taken up into neurons where they template endogenous tau to take on the abnormal conformation, resulting in pathology (Guo and Lee, 2011; Guo et al., 2016; Narasimhan et al., 2017; Xu et al., 2021). Tau seeds isolated from the brains of AD patients consists of both 3R and 4R tau, and as such the presence of both 3R and 4R tau in MAPT KI mice provides substrate to template to the abnormally folded 3R + 4R AD-tau seeds. There appears to be an asymmetric barrier to cross seeding between 3R and 4R tau, where misfolded 4R tau is only able to recruit 4R tau into aggregates (Dinkel et al., 2011; Kumar and Udgaonkar, 2018). Thus, the 3R + 4R tau in MAPT KI mice can template both isoforms of misfolded tau found in tau seeds derived from AD, as well as other human tauopathies with primarily 3R or 4R pathology. Indeed, the humanization of the murine *Mapt* gene accelerates tauopathy induced by the injection of AD-tau seeds (Saito et al., 2019). Finally, the lack of a tau-based phenotype through 24 m of age in MAPT KI mice supports the utility of this model to test upstream factors suspected of initiating tau pathology and toxicity. Crossing APP^NL-G-F^ KI mice with MAPT KI mice enhanced abnormal tau phosphorylation and dystrophic neurites compared to APP^NL-G-F^ KI mice (Saito et al., 2019). More recently, the Goedert group showed that the APP^NL-G-F^/mutant P290S MAPT double KI mice produced a more robust tau pathology phenotype than P290S MAPT single KI mice (Huang et al., 2022). These results demonstrate the utility of MAPT KI models to studying modifiers of tau pathology and other suspected initiating factors should be investigated with these models. Taken together, the lack of tau pathology and the expression of both 3R and 4R tau isoforms makes MAPT KI mice ideal models for future studies aiming to define the precise consequences of tauopathy induced by the injection of pathological tau seeds or upstream pathology-initiating factors.

In conclusion, we have provided a detailed characterization showing that MAPT KI mice express all 6 human tau isoforms at physiological levels in the absence of behavioral or neuropathological abnormalities. Further, MAPT KI mice show signs consistent with normal brain aging in healthy individuals. The data presented herein can serve as a baseline for future studies utilizing this model to study the normal function of tau as well as the consequences of tau dysfunction.

## Data availability statement

The original contributions presented in the study are included in the article/Supplementary material, further inquiries can be directed to the corresponding author.

## Ethics statement

The animal study was approved by Michigan State University Institutional Animal Care and Use Committee. The study was conducted in accordance with the local legislation and institutional requirements.

## Author contributions

MB: Conceptualization, Data curation, Formal analysis, Investigation, Methodology, Validation, Visualization, Writing – original draft, Writing – review & editing. SP: Formal analysis, Investigation, Methodology, Writing – review & editing. TS: Resources, Writing – review & editing. TCS: Resources, Writing – review & editing. TG: Formal analysis, Investigation, Methodology, Writing – review & editing. NK: Conceptualization, Data curation, Formal Analysis, Funding acquisition, Methodology, Project administration, Resources, Supervision, Validation, Visualization, Writing – original draft, Writing – review & editing.

## Figures and Tables

**Table 1:** 

MAPT KI	Human MAPT knock-in
WT	Wild type
NeuN	Neuronal Nuclei
GAPDH	Glyceraldehyde phosphate dehydrogenase
MAP2	Microtubule association protein 2
PSD-95	Post synaptic density 95
IBA1	Ionized calcium adaptor molecule 1
GFAP	Glial fibrillary acidic protein
MBP	Myelin basic protein
M	Months
MAP	Microtubule associated protein
AD	Alzheimer’s disease
PSP	Progressive supranuclear palsy
CBD	Corticobasal degeneration
FTD-17	Frontotemporal dementia linked to chromosome 17
LTD	Long term depression
NFT	Neurofibrillary tangle
