# Supplementary material for: Behavioral and neuropathological characterization over the adult lifespan of the human tau knock-in mouse
Source: Front Aging Neurosci. 2023 Sep 29;15:1265151. doi: 10.3389/fnagi.2023.1265151 (PMC10576558; doi:10.3389/fnagi.2023.1265151)
Supplement: Supplementary file 2 [file Data_Sheet_1.docx]

**Supplemental Figures and Figure Legends**

**Behavioral and Neuropathological Characterization Over the Adult Lifespan of the Human Tau Knock-in Mouse**

***Matthew J. Benskey^1^, Spencer Panoushek^1^, Takashi Saito^3,4^, Takaomi C. Saido^4^, Tessa Grabinski^1^, Nicholas M. Kanaan^1,2^.***

^1^Department of Translational Neuroscience, College of Human Medicine, Michigan State University, Grand Rapids, MI

^2^Neuroscience Program, Michigan State University, East Lansing, MI 48824

^3^Department of Neurocognitive Science, Institute of Brain Science, Nagoya City University Graduate School of Medical Sciences, Nagoya, Aichi, Japan

^4^Laboratory for Proteolytic Neuroscience, Riken Center for Brain Science, Wako, Saitama, Japan

**Supplemental Figure 1**


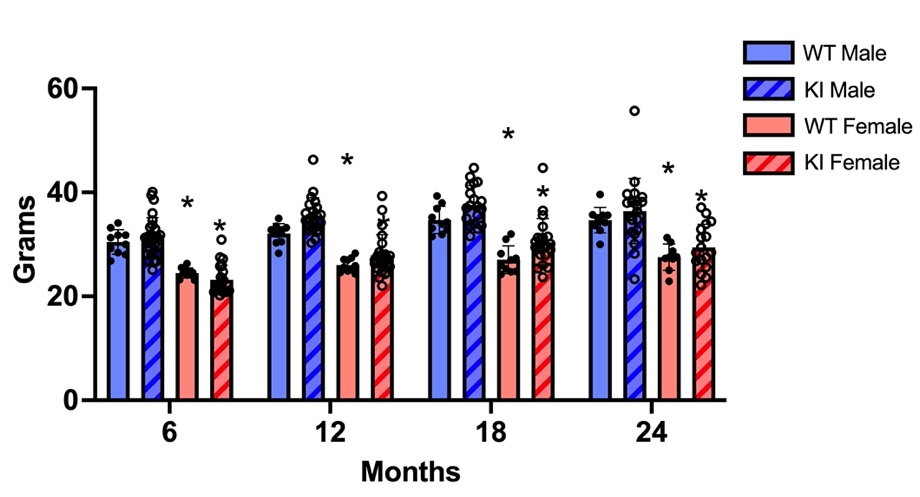


**Supplemental Figure 1. Animal Weights.** Male and female human MAPT knock-in (KI) and wild type (WT) mice were weighed prior to sacrifice at 6, 12, 18 and 24m of age. Individual weights (in grams) are shown while bars represent the mean of groups ± standard deviation. *Significantly different than male group in same age and genotype (p ≤ 0.05 (n = 10-20/group).

**Supplemental Figure 2**

**
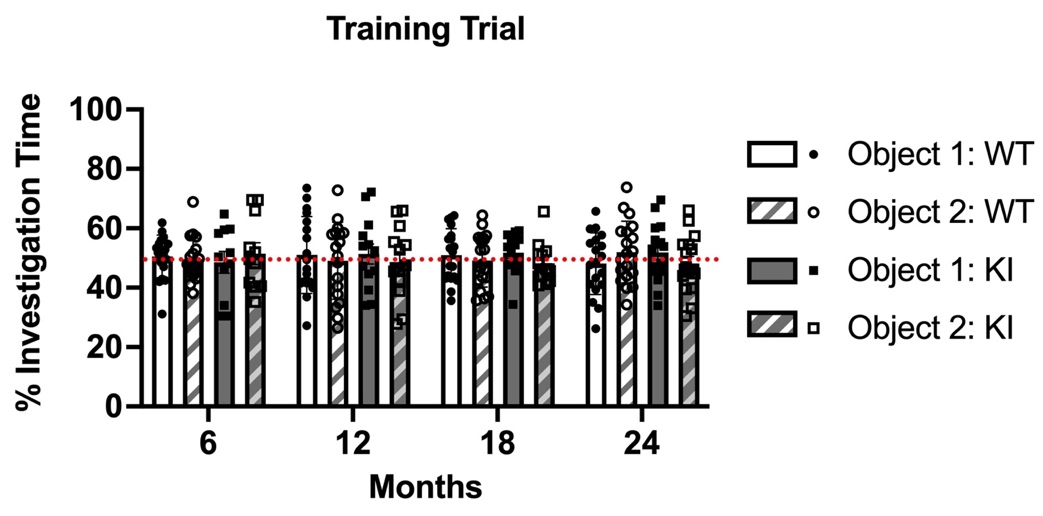
**

**Supplemental Figure 2. MAPT KI and WT Mice Do Not Display Preference Towards Objects Used in the OLT-NORT.** Male and female human MAPT knock-in (MAPT KI or KI) and wild type (WT) mice completed the object location task (OLT) and novel object recognition task (NORT) at 6, 12, 18, and 24 months (m) of age (n = 6-10/group). For all tasks, no sex differences were detected within a given age and genotype (see **Supplemental Table 1** for data on individual sexes). Sexes were combined within age and genotype for all subsequent analyses. Prior to the OLT-NORT trials, mice were trained to recognize two identical objects in trial 1. Quantification of the percent of time spent investigating each object is shown. There were no differences in the amount of time any group spent investigating objects during training in trial 1, confirming a lack of bias towards either of the objects used or the respective sides of the open field apparatus where the assay was performed.

**Supplemental Figure 3**

**
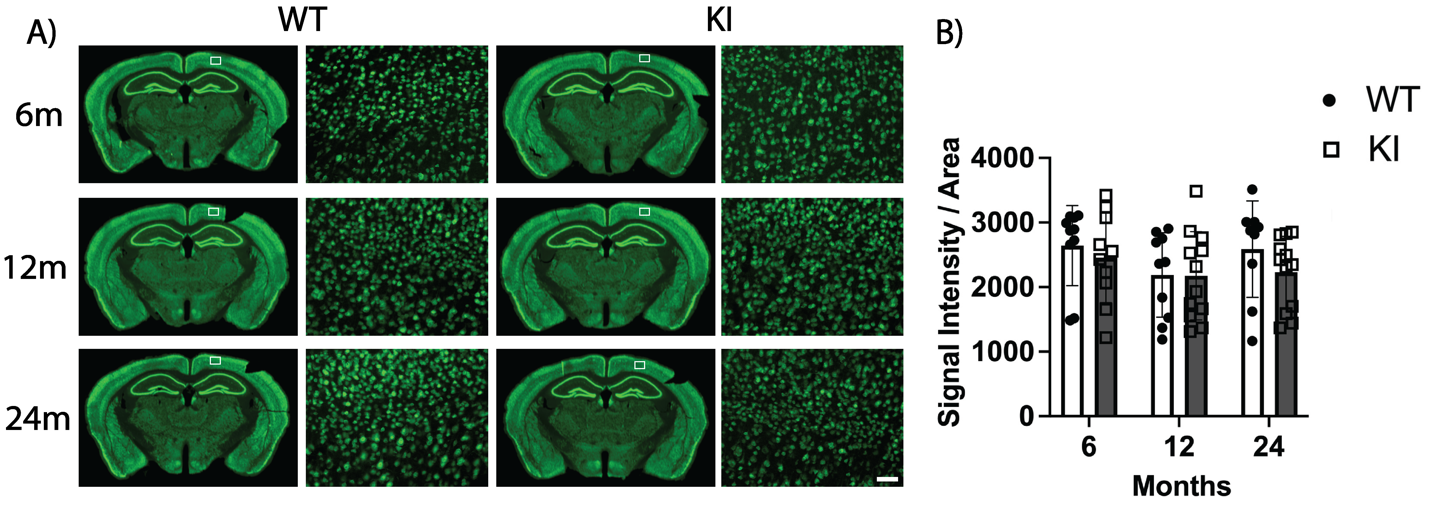
**

**Supplemental Figure 3. Quantification of Cortical NeuN in MAPT KI Mice.** Tissue from 6-, 12- and 24-months old male and female human MAPT knock-in (MAPT KI or KI) and wild type (WT) mice (n = 4-5/group) was processed to perform immunofluorescence detection of the marker of neuronal nuclei (NeuN) in the cortex. For all endpoints, no sex differences were detected within a given age and genotype (see **Supplemental table 2.1A-2.3A** for data on individual sexes). Sexes were combined within age and genotype for all subsequent comparisons. A) Representative images of NeuN immunofluorescence in the cortex of MAPT KI and WT mice. High magnification images of NeuN immunofluorescence correspond to the area within in the box in the low magnification images. B) Quantification of NeuN fluorescence intensity normalized to the area of the respective hippocampal sections. For all histograms, individual data points are shown while bars represent the mean of groups ± standard deviation (n = 8-10/group for combined sexes). Scale bar in the lower right panel of (A) is 50 μm and applies to all other high magnification panels.

**Supplemental Figure 4**


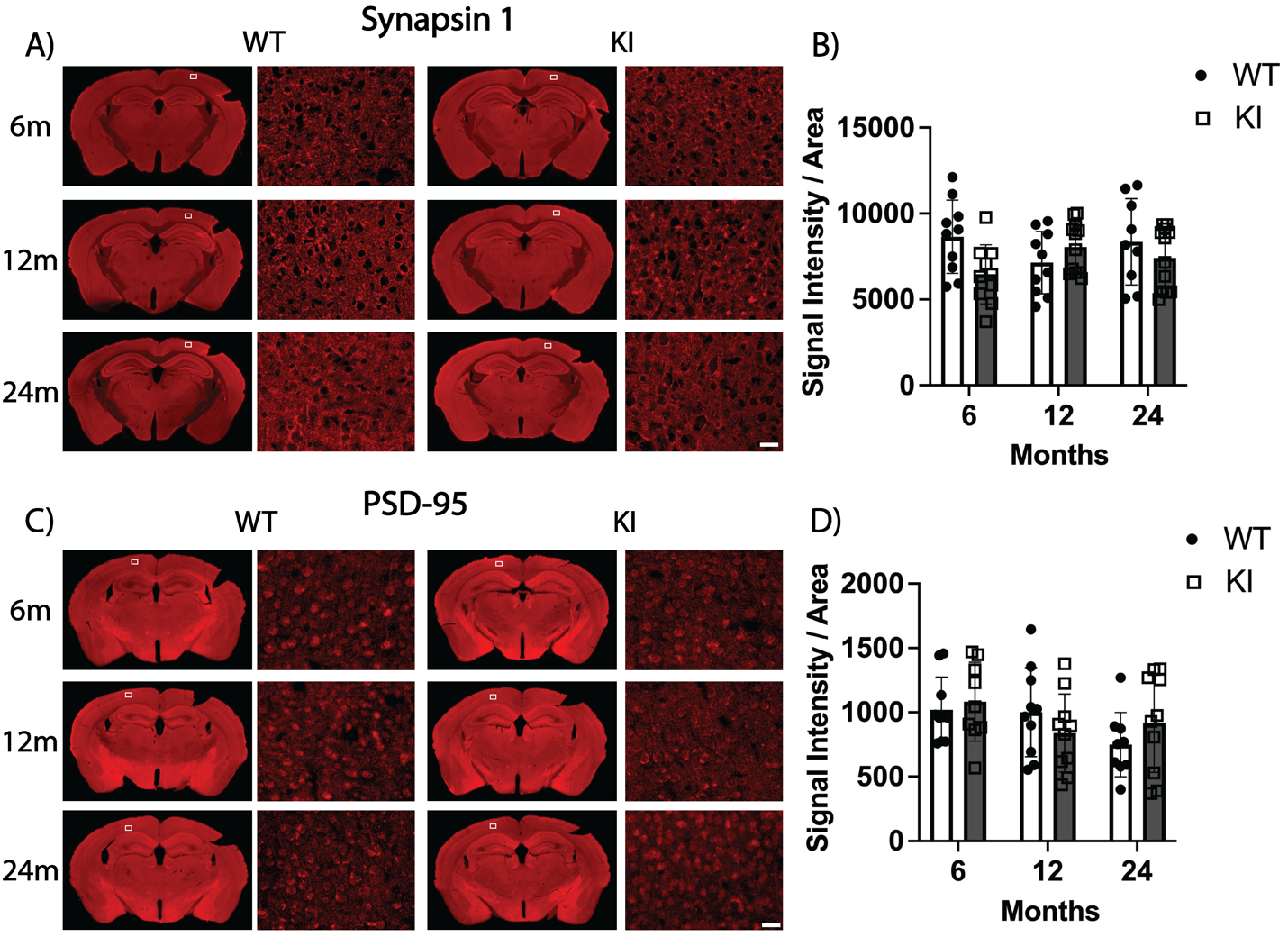


**Supplemental Figure 4. Quantification of Cortical Synaptic Markers in MAPT KI Mice.** Tissue from 6-, 12- and 24-months old male and female human MAPT knock-in (MAPT KI or KI) and wild type (WT) mice (n = 4-5/group) was processed for immunofluorescence detection of the presynaptic marker synapsin 1 (A-B) and the post synaptic marker PSD-95 (C-D) in the cortex. For all endpoints, no sex differences were detected within a given age and genotype (see **Supplemental Table 2.4A-2.5A** for data on individual sexes). Sexes were combined within age and genotype for all subsequent comparisons. A) Representative images of synapsin 1 immunofluorescence in MAPT KI and WT mice. High magnification images of synapsin 1 immunofluorescence correspond to the area within in the box in the low magnification images. B) Quantification of synapsin 1 fluorescence intensity normalized to the area of the respective cortical sections. C) Representative images of PSD-95 immunofluorescence in MAPT KI and WT mice. High magnification images of PSD-95 immunofluorescence correspond to the area within in the box in the low magnification images. D) Quantification of PSD-95 fluorescence intensity normalized to the area of the respective cortical sections. For all histograms, individual data points are shown while bars represent the mean of groups ± standard deviation (n = 8-10/group for combined sexes). Scale bars in the lower right panel of A and B are 50 μm and apply to all other high magnification panels.

**Supplemental Figure 5**


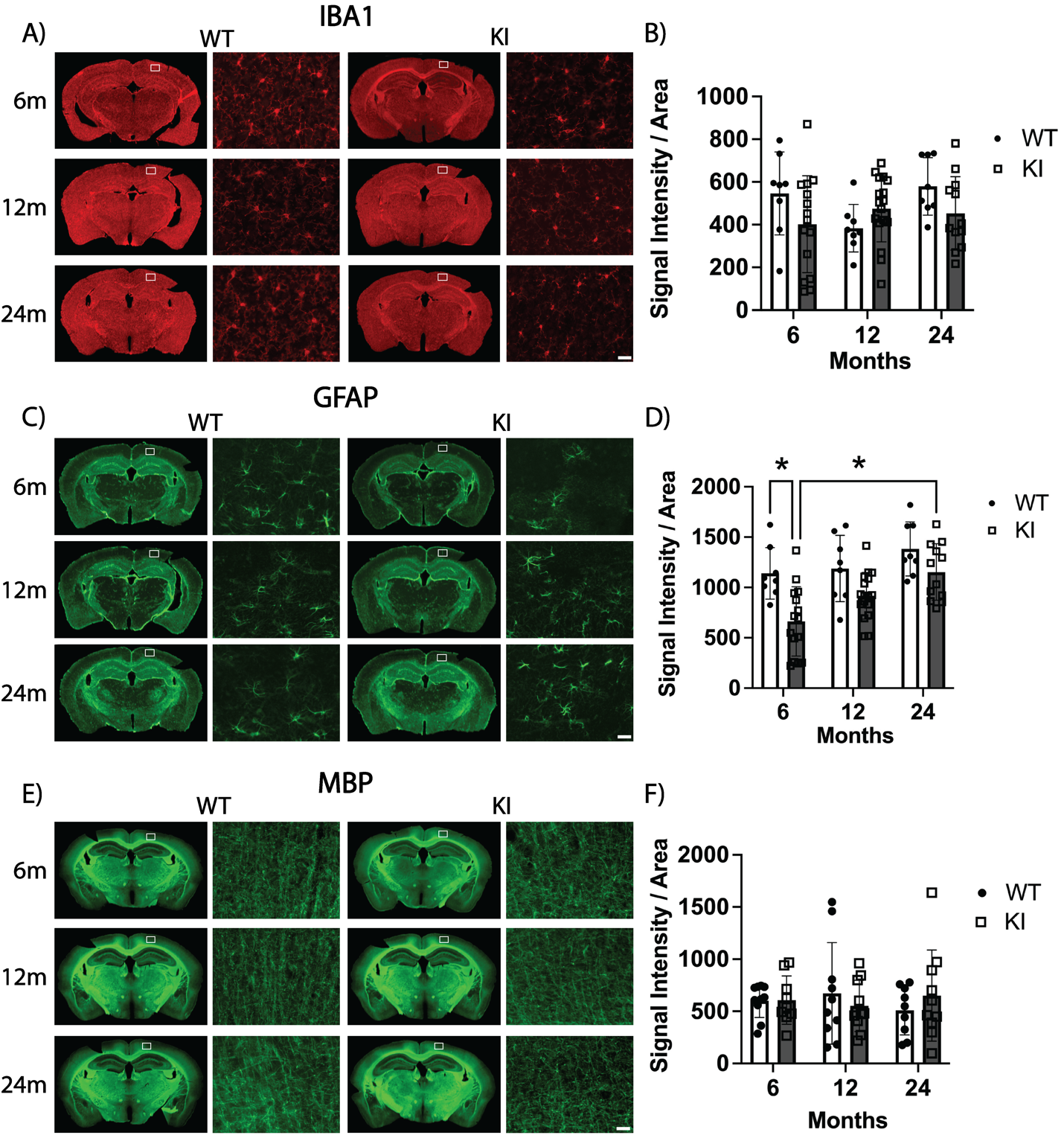


**Supplemental Figure 5. Quantification of Cortical IBA1, GFAP, and MBP in MAPT KI Mice.** Tissue from 6, 12 and 24 months old male and female human MAPT knock-in (MAPT KI or KI) and wild type (WT) mice (n = 4-5/group) was processed to perform immunofluorescence detection of the microglial marker ionized calcium adapter molecule 1 (IBA1; panels A-B), the astrocytic marker glial fibrillary acidic protein (GFAP; panels C-D) and the oligodendrocyte protein, myelin basic protein (MBP; panels E-F) in the cortex. For all endpoints, no sex differences were detected within a given age and genotype (see **Supplemental table 2.6A, 2.7A, 2.8A** for data on individual sexes). Sexes were combined within age and genotype for all subsequent comparisons. A) Representative images IBA1 immunofluorescence in MAPT KI and WT mice. High magnification images of IBA1 immunofluorescence correspond to the area within in the box in the low magnification images to the left of each panel. B) Quantification of IBA1 fluorescence intensity normalized to the area of the respective cortical sections. C) Representative low magnification images of GFAP immunofluorescence in MAPT KI and WT mice. High magnification images of GFAP immunofluorescence correspond to the area within in the box in the low magnification images. D) Quantification of GFAP fluorescence intensity normalized to the area of the respective cortical sections. E) Representative low magnification images of MBP immunofluorescence in MAPT KI and WT mice. High magnification images of MBP immunofluorescence correspond to the area within in the box in the low magnification images. F) Quantification of MBP fluorescence intensity normalized to the area of the respective cortical sections. For all histograms, individual data points are shown while bars represent the mean of groups ± standard deviation (n = 8-10/group for combined sexes). Scale bars in the lower right panel of A, C, and E represent 50 μm and apply to all other high magnification panels. * p ≤ 0.05 (n = 6-8/group for combined sexes).

**Supplemental Figure 6**


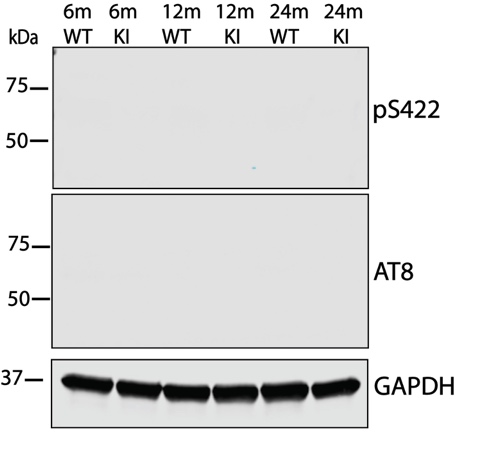


**Supplemental Figure 6. pS422 and AT8 Phosphorylated Tau are Absent in the Hippocampus of MAPT KI mice.** Fresh tissue from 6, 12 and 24 months (m) old male and female human MAPT knock-in (MAPT KI or KI) and wild type (WT) mice (n = 4-5/group) was processed to perform immunoblotting for detection of AT8 (phosphorylation within aa198-210) and pS422 (Ser 422) and glyceraldehyde phosphate dehydrogenase (GAPDH). Representative blots show no AT8 or pS422 reactivity in the Hp of MAPT KI and WT mice.
